# Machine learning-based integration develops an immune-related risk model for predicting prognosis of high-grade serous ovarian cancer and providing therapeutic strategies

**DOI:** 10.3389/fimmu.2023.1164408

**Published:** 2023-04-05

**Authors:** Qihui Wu, Ruotong Tian, Xiaoyun He, Jiaxin Liu, Chunlin Ou, Yimin Li, Xiaodan Fu

**Affiliations:** ^1^Department of Gynecology, Xiangya Hospital, Central South University, Changsha, China; ^2^National Clinical Research Center for Geriatric Disorders, Xiangya Hospital, Changsha, China; ^3^Department of Pharmacology, School of Basic Medical Sciences, Shanghai Medical College, Fudan University, Shanghai, China; ^4^Departments of Ultrasound Imaging, Xiangya Hospital, Central South University, Changsha, Hunan, China; ^5^Department of Pathology, School of Basic Medical Sciences, Central South University, Changsha, China; ^6^Department of Pathology, Xiangya Hospital, Central South University, Changsha, China; ^7^Department of Pathology, Fudan University Shanghai Cancer Center, Shanghai, China; ^8^Department of Oncology, Shanghai Medical College, Fudan University, Shanghai, China

**Keywords:** tumor microenvironment, ovarian cancer, machine learning, prognosis, treatment

## Abstract

**Background:**

High-grade serous ovarian cancer (HGSOC) is a highly lethal gynecological cancer that requires accurate prognostic models and personalized treatment strategies. The tumor microenvironment (TME) is crucial for disease progression and treatment. Machine learning-based integration is a powerful tool for identifying predictive biomarkers and developing prognostic models. Hence, an immune-related risk model developed using machine learning-based integration could improve prognostic prediction and guide personalized treatment for HGSOC.

**Methods:**

During the bioinformatic study in HGSOC, we performed (i) consensus clustering to identify immune subtypes based on signatures of immune and stromal cells, (ii) differentially expressed genes and univariate Cox regression analysis to derive TME- and prognosis-related genes, (iii) machine learning-based procedures constructed by ten independent machine learning algorithms to screen and construct a TME-related risk score (TMErisk), and (iv) evaluation of the effect of TMErisk on the deconstruction of TME, indication of genomic instability, and guidance of immunotherapy and chemotherapy.

**Results:**

We identified two different immune microenvironment phenotypes and a robust and clinically practicable prognostic scoring system. TMErisk demonstrated superior performance over most clinical features and other published signatures in predicting HGSOC prognosis across cohorts. The low TMErisk group with a notably favorable prognosis was characterized by BRCA1 mutation, activation of immunity, and a better immune response. Conversely, the high TMErisk group was significantly associated with C-X-C motif chemokine ligands deletion and carcinogenic activation pathways. Additionally, low TMErisk group patients were more responsive to eleven candidate agents.

**Conclusion:**

Our study developed a novel immune-related risk model that predicts the prognosis of ovarian cancer patients using machine learning-based integration. Additionally, the study not only depicts the diversity of cell components in the TME of HGSOC but also guides the development of potential therapeutic techniques for addressing tumor immunosuppression and enhancing the response to cancer therapy.

## Introduction

Although targeted drugs for ovarian cancer (OC), consisting of PARP inhibitors and Bevacizumab, limitedly prolong the survival of patients with advanced disease, OC continues to be the leading cause of cancer death in women (1). Among multiple histological types of epithelial OC, high-grade serous ovarian cancer (HGSOC) is the most common type, accounting for 60–80% of all cases and being responsible for approximately 80% of all OC deaths (2, 3). For more precise clinical management of patients, the researchers devote themselves to investigating the subtypes of OC, including The Cancer Genome Atlas (TCGA) project, generally suggesting that, in addition to molecular subtypes of tumor cells with different mutations or abnormal activation states, heterogeneity in proportion and anatomical location of non-tumor cells also leads to different phenotypes (4–6). Indeed, molecular or immunological subtyping gives unique insights for basic research, but the robustness of these subtypes across studies and their clinical implications remain controversial (7).

Inarguably, cancer immunotherapies, including immune checkpoint blockade (ICB), have significantly improved the treatment of advanced solid tumors and benefited overall survival of patients when compared to conventional therapy (8, 9). However, in patients with advanced ovarian cancer, the benefit of ICBs is limited (10, 11). The mortality rate continues to be a growing concern. Despite repeated associations between HGSOC survival and T cell infiltration, especially for tumor-infiltrating CD8+ T lymphocytes (TILs), human HGSOC remains poorly responsive to immunotherapy (12, 13). One possible explanation for this failure is that T cells are unable to penetrate the extracellular matrix (14, 15).

It is now well recognized that the TME, the soil in which tumors live and thrive, influences prognosis and therapy effectiveness. With advances in single-cell sequencing, depicting the cellular diversity in the TME at high resolution provides a characterization of the cellular composition in three tumor immune phenotypes (infiltrated, excluded, and desert) in HGSOC (16) and highlight the contributions of tumor-associated stromal components in supporting tumor growth and hindering the efficacy of immunotherapy (4, 17, 18).

While previous studies have explored the potential of constructing gene signatures based on TME or immune-related genes as predictive indicators for tumor prognosis and immunotherapy (19, 20). The published studies have limited predictive performance when assessed in different independent cohorts. In this study, mining data from several HGSOC bulk RNA-seq datasets, we aim to uncover the TME subtypes in HGSOC with consistency across multiple datasets and develop a robust and clinically practicable prognostic scoring system. Considering the contributions of both immune and stromal components, we start with identifying the inherent TME subtypes from a meta-cohort of HGSOC and TME-related genes. To address the robustness of the scoring system, we have implemented 108 combination frames constructed by 10 machine learning algorithms to achieve the best prognostic scoring performance that was assessed in multiple independent cohorts. The scoring system was termed the TMErisk score, which was able to indicate genomic instability, recognize the tumor immune microenvironment and cancer-related dysfunctions, and guide the identification of effective treatments for individual HGSOC patients.

## Materials and methods

### Data collection and processing

This study included seven public cohorts of HGSOC tumors, including two RNA-Seq datasets from the International Cancer Genome Consortium (ICGC; OV-AU) portal and The Cancer Genome Atlas (TCGA; TCGA-OV), as well as five microarray datasets from the Gene Expression Omnibus (GEO: GSE13876, GSE140082, GSE30161, GSE32062, and GSE9891) (**Table S1**). Besides the transcript data, the corresponding clinical data was also taken into account. A total of 1386 HGSOC tumor samples were included in this study, which excluded patients whose overall survival data was insufficient. From the Genotype-Tissue Expression Database (GTEx, https://gtexportal.org/home/), the expression information of normal ovarian samples was downloaded. The IMvigor210 cohort, an immune checkpoint blockade treatment cohort, was obtained from http://research-pub.gene.com/IMvigor210CoreBiolo. These cohorts’ initial raw data were pre-processed and normalized in accordance with our previous studies (19, 21). As for RNA-Seq datasets, raw counts were converted into values for the number of transcripts per million bases (TPM). The batch effects among various cohorts were eliminated using the “ComBat” algorithm of the “SVA” package. Somatic mutations and copy number variations (CNAs) of HGSOC were downloaded from TCGA. The mutation landscape of TCGA-OV was examined and represented using the “maftools” and “ComplexHeatmap” packages. Fisher’s exact test was used to identify the top 20 mutation genes and differentially mutated genes. A GISTIC 2.0 analysis was conducted to investigate the CNV associated with HGSOC by GenePattern (https://www.genepattern.org/).

### Human tissue specimens

In this study, a total of 25 patients with high-grade serous ovarian cancer (HGSOC) who had undergone curative resection at Xiangya Hospital, Central South University, were recruited. All patients provided informed consent, and the study was approved by the Xiangya Hospital Ethics Committee.

### RNA extraction and real-time quantitative PCR

Total RNA was extracted from human tissue specimens using FFPE RNA Extraction Kits (AmoyDx, Xiamen, China), in accordance with the manufacturer’s instructions. The purity and quantity of RNA were evaluated using the NanoDrop 1000 Spectrophotometer (Thermo Fisher, USA), with OD260/OD280 ratios of 1.8-2.0 and OD260/230 ratios of 2.0-2.2. Reverse transcription was carried out using HiScript II Reverse Transcriptase (Vazyme, Nanjing, China) from 1 μg of total RNA, to obtain first-strand cDNA. Quantitative real-time PCR (qRT-PCR) was conducted in triplicate on an ABI Prism 700 thermal cycler (Applied Biosystems, Foster City, CA, USA), as previously described (22). GAPDH was used as the reference gene for RNA quantification. The following primer sequences were used: SNRPE (forward primer: ATGTCAGGACTAGGAGCCACTGTG; reverse primer: AGCATGATCCGACCCAGTTGTTTTC), CD274 (forward primer: GACCACCACCACCAATTCCAAGAG; reverse primer: TGAATGTCAGTGCTACACCAAGGC), CD8A (forward primer: GCGAGACAGTGGAGCTGAAGTG; reverse primer: ACGAAGTGGCTGAAGTACATGATGG), and GAPDH (forward primer: AACGGATTTGGTCGTATTGG; reverse primer: TTGATTTTGGAGGGATCTCG).

### Estimation of TME cell infiltration

The relative abundance of immune and stromal cells infiltrated in the TME of HGSOC was quantified using the “XCELL” package in accordance with the gene expression profiles. In this study, the abundance of CD8+ T cells and the ESTIMATE score were calculated using the ssGSEA, EPIC, TIMER, QUANTISEQ, MCPCOUNTER, XCELL, CIBERSORT, CIBERSORT-ABS, and ESTIMATE algorithms (23–29).

### Identification of ovarian cancer TME-related genes

Consensus clustering was carried out using the “ConsensusClusterPlus” package to identify TME-related subtypes for additional investigation in accordance with the infiltration of immune and stromal cells (30). Using the limma package (31), the differentially expressed genes (DEGs) between various immune subtypes were screened with an adjusted *P* < 0.05. TME-related genes of HGSOC were defined as genes that are co-upregulated or co-downregulated in not fewer than six cohorts.

### Construction of the TMErisk score

We used the same procedures as in the previous study to screen out the most valuable TMErisk score (32, 33). First, ovarian cancer TME-related genes in each cohort were subjected to univariate Cox regression analysis. Genes with a stable prognostic value were then further filtered out, with the filter criteria being an adjusted *P* < 0.1 and the same hazard ratio direction for at least five cohorts. Second, a machine learning-based integrative method was developed using ten distinct machine learning algorithms, including Lasso, Ridge, stepwise Cox, CoxBoost, random survival forest (RSF), elastic network (Enet), partial least squares regression for Cox (plsRcox), supervised principal components (SuperPC), generalized boosted regression modeling (GBM), and survival support vector machine (survival-SVM). Then, to fit the most useful prediction models in the TCGA-OV cohort, 108 algorithm combinations from 10 machine learning algorithms were applied to the TME- and prognostic-related genes. Each of these prediction models was further tested in validation cohorts, and the C-index was calculated for each cohort. Finally, the TMErisk signature was built using the CoxBoost and SuperPC algorithms, which had the highest average C-index in the validation cohorts.

### Pathway enrichment analysis

The R package “clusterProfiler” was used to conduct analyses of the Gene Ontology (GO), Kyoto Encyclopedia of Genes and Genomes (KEGG), and gene set enrichment analysis (GSEA) (34). With the “GSVA” package, single-sample GSEA (ssGSEA) was also carried out (35).

### Prediction of response to immunotherapy or chemotherapy

Different tumor immune evasion mechanisms were modeled using the Tumor Immune Dysfunction and Exclusion (TIDE) algorithm (36). Immunophenoscore (IPS) and Subclass mapping were used to predict anti-PD-1 and anti-CTLA-4 immunotherapy responses between low- and high-TMErisk groups (37). Based on the genomics of drug sensitivity in cancer, the ridge regression model implemented in the “pRRophetic” package was chosen to predict the chemotherapy response of each sample (38). To find potential therapeutic agents, Spearman correlation analysis and differential analysis between various TMErisk groups were conducted.

### Statistical analysis

All data processing was carried out using R 4.0.5 software. To compare continuous variables, the Wilcoxon and Kruskal-Wallis tests were used, and the chi-square (χ2) test was used to test categorical data. Correlation coefficients were calculated using Spearman’s correlation test. To examine any associated independent predictors of prognosis in HGSOC, the log-rank test, univariate, and multivariable Cox regression models were used. Statistical significance was defined as a two-sided *P* value < 0.05.

## Results

### Consensus clustering for TME-infiltrating cells

**Figure 1:**
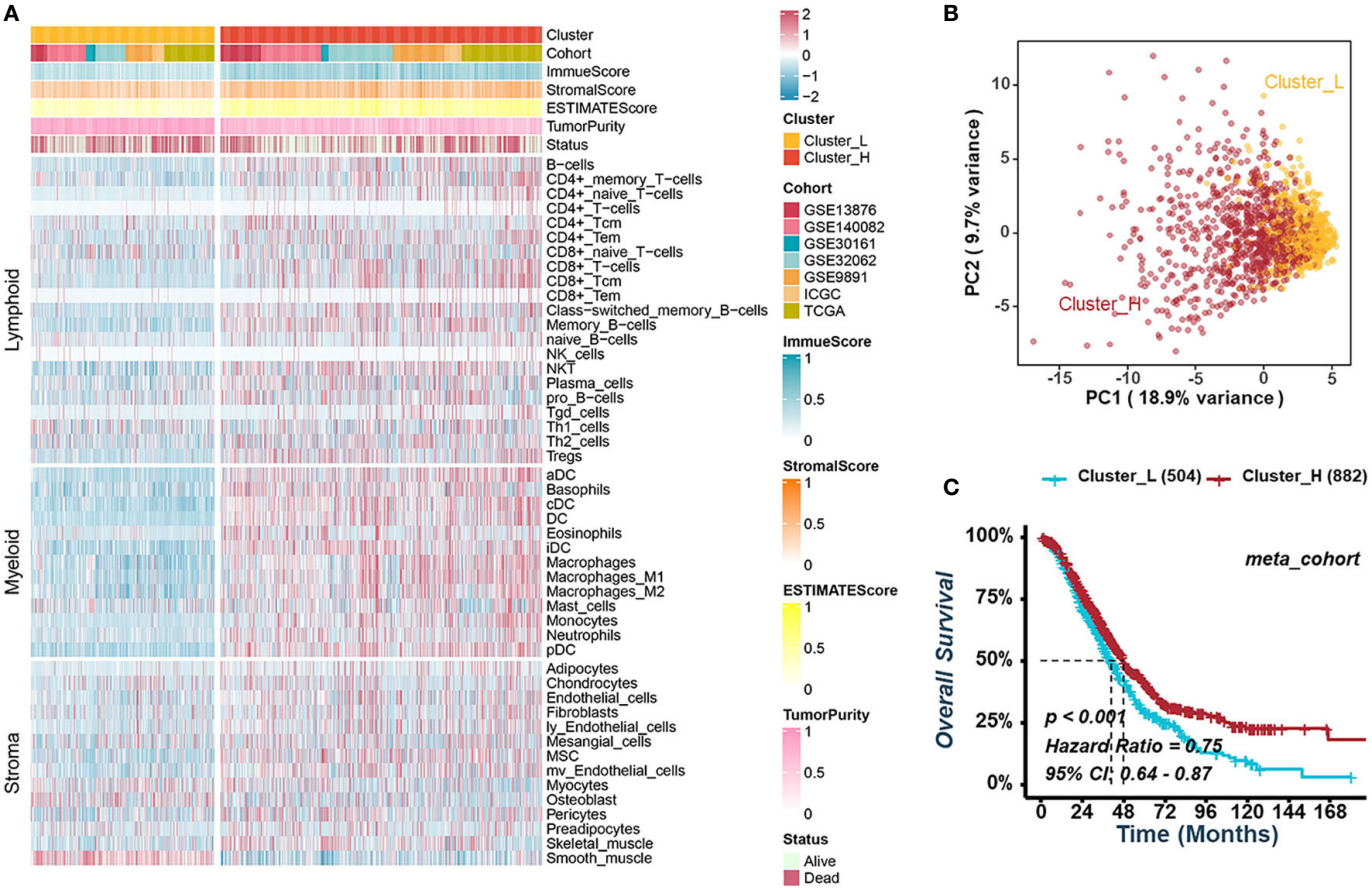
Consensus clustering for TME-infiltrating cells in HGSOC. **(A)** Heatmap illustrating the infiltration of immune and stromal cells between clusters-L and cluster-H in the meta-cohort. **(B)** Principal component analysis suggesting two distinct clusters in the meta-cohort. **(C)** Kaplan-Meier analysis estimating the overall survival between cluster-L and cluster-H.

The TME of HGSOC requires consideration of more than just immune cells due to its significant stromal characteristics. To identify potential tumor-immune-stroma phenotypes of HGSOC, we performed consensus cluster analyses in seven independent cohorts (GSE13876, GSE140082, GSE30161, GSE32062, GSE9891, ICGC, and TCGA-OV) and an integrated meta-cohort based on 48 signatures of non-tumor components in TME, including lymphocytes, myeloid, and stromal cells (**Figure 1A**, **Figure S1**). As shown in **Figures S1A, B**, two clusters could achieve the best clustering efficacy in the meta-cohort, and similar clustering results were obtained in all seven independent cohorts (**Figures S1C–I**). The result of clustering demonstrated that the distribution of cell signatures was biased between the two clusters. Thus, we defined the cluster with higher infiltration of immune and stromal cells as cluster-H and named the cluster with lower ones as cluster-L (**Figure 1A**). Principal component analysis (PCA) suggested a significant difference between the two clusters (**Figure 1B**). Survival analyses indicated that cluster-H was correlated with a notably favorable prognosis in GSE13876 (log-rank test, *P* = 0.003), GSE32062 (log-rank test, *P* = 0.011), and the meta-cohort (log-rank test, *P* < 0.001), while there was no significant correlation in the other cohorts (**Figures 1C**, **S2**).

### Construction of the TME-related risk score in HGSOC

**Figure 2:**
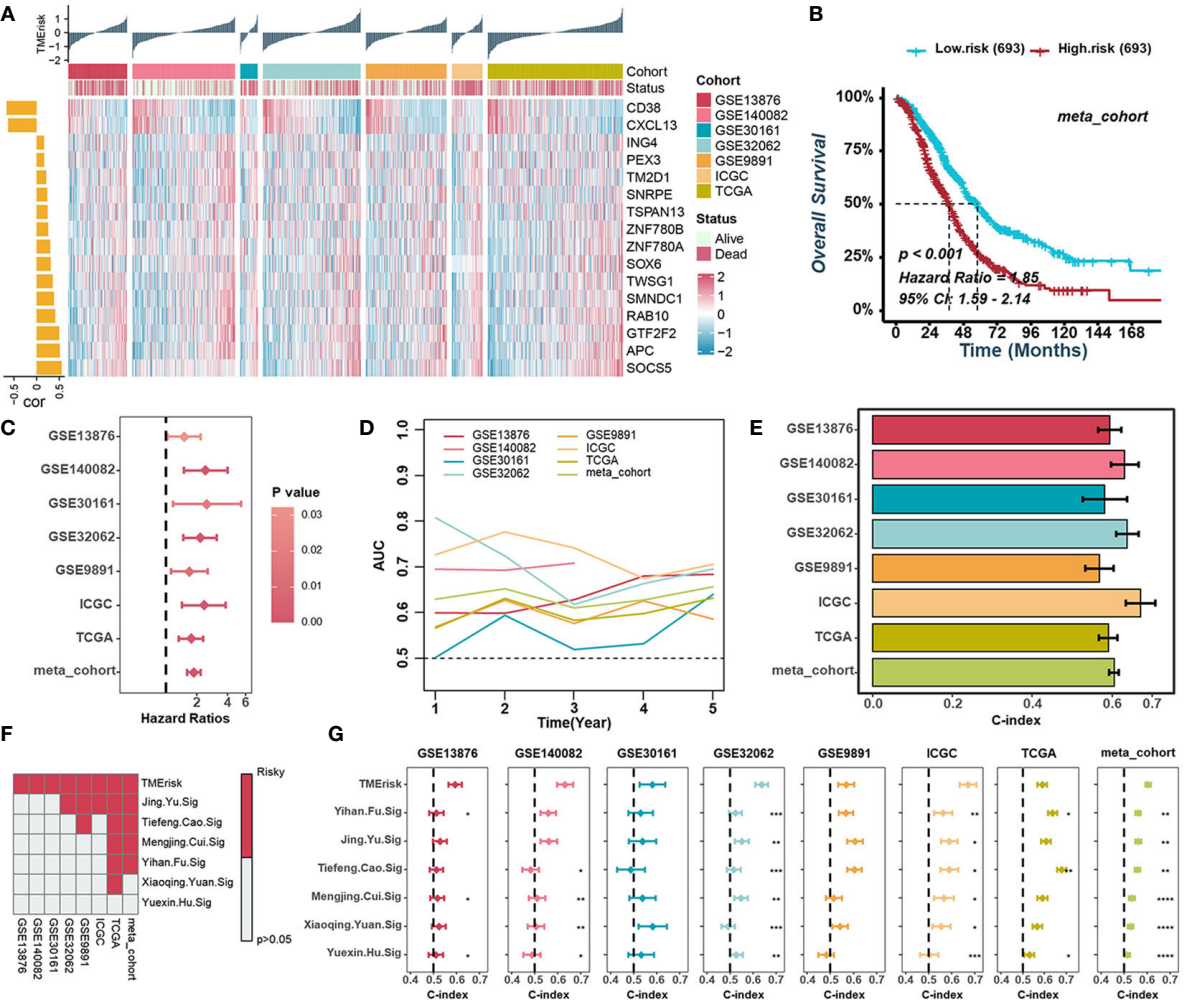
Construction of the TMErisk score in HGSOC. **(A)** Heatmap illustrating the expression of 16 TME-related genes and the TMErisk score in low- and high-TMErisk groups. The bar chart on the left illustrates the relationships between TME-related genes and TMErisk score. **(B)** Kaplan-Meier analysis estimating the overall survival between low- and high-TMErisk groups in meta-cohort. **(C)** Univariate Cox regression analyses revealing the correlation between TMErisk score and HGSOC survival. **(D)** Time-dependent AUC value of the TMErisk score in different cohorts. **(E)** C-index of the TMErisk score in different cohorts. **(F)** Univariate Cox regression analysis of the TMErisk score and other published signatures across diverse cohorts. **(G)** C-index of the TMErisk score and other published signatures across diverse cohorts. *P < 0.05; **P < 0.01; ***P < 0.001; ****P < 0.0001.

To mine for TME-related genes specific to HGSOC, we screened out the differentially expressed genes (DEGs) between Cluster-H and Cluster-L with an adjusted *P* < 0.05 in all cohorts. The gene that was upregulated or downregulated in no less than six cohorts was defined as TME-related genes of HGSOC for further integrated analysis. In Cluster-H, there were 390 upregulated genes and 1260 downregulated genes, respectively (**Figures S3A, B**). Gene Ontology (GO) and Kyoto Encyclopedia of Genes and Genomes (KEGG) pathway enrichment analyses indicated that the upregulated genes in Cluster-H were mainly enriched in immune-related signatures, indicating the reliable results we obtained before (**Figures S3C–F**). Subsequently, univariate Cox regression analysis was performed on TME-related genes, and 76 genes had stable prognostic value in different cohorts (**Figure S4A**). These 76 genes associated with prognosis were included in the procedures based on different combinations of machine learning algorithms to develop a TME-related risk score (TMErisk). As in the previous study by Zaoqu Liu et al. (32), we integrated 10 machine learning algorithms, including CoxBoost, stepwise Cox, Ridge, RSF, GBM, survival-SVM, Lasso, Enet, plsRcox, and SuperPC, to acquire the TMErisk with high accuracy and stability performance in different cohorts. In the TCGA-OV cohort, 108 kinds of prediction models were fitted, and the average C-index of each model in the other seven validation cohorts was further calculated (**Figure S4B**). Among these prediction models, a combination of CoxBoost and SuperPC algorithms had the highest average C-index in validation cohorts, and the 16 most valuable TME-related genes (APC, CD38, CXCL13, GTF2F2, ING4, PEX3, RAB10, SMNDC1, SNRPE, SOCS5, SOX6, TM2D1, TSPAN13, TWSG1, ZNF780A, and ZNF780B) were identified by the CoxBoost algorithm (**Figure 2A**, **S4B**). CD38 and CXCL13 were negatively correlated with the TMErisk score, while the others were positively correlated with the TMErisk score (**Figure 2A**). Also, we compared the expression of 16 TME-related genes between HGSOC (TCGA-C) and normal ovarian tissue (GTEx-N) and noticed that CD38, CXCL13, RAB10, SNRPE, SOX6, TSPAN13, and TWSG were upregulated in HGSOC, while APC, GTF2F2, SOCS5, TM2D1, ZNF780A, and ZNF780B were downregulated in HGSOC (**Figure S5A**).

### Prognostic value of the TMErisk score in HGSOC

It should be considered that TME in HGSOC patients is not only determined by the type of cells infiltrated but also by the molecular characteristics of the tumor and the individual conditions of the patients. Therefore, we examined the scores in different types of patient groups. There was no difference in TMErisk scores between age, grade, or stage subgroups. TMErisk score did, nevertheless, correlate with immune and molecular subtypes (**Figure S6A**). Specifically, the TMErisk scored highest in the proliferative molecular subtype and lowest in the immunoreactive and IFN-dominant subtypes (**Figure S6A**). Meanwhile, the cluster-L group had a higher TMErisk score (**Figure S6A**). According to the median TMErisk score in each cohort, HGSOC patients were divided into high- or low- TMErisk groups. Kaplan–Meier survival analyses exhibited that the patients in the high TMErisk score group had poorer overall survival in all cohorts, and unfavorable progression-free survival in six cohorts (**Figures 2B**, **S7A, B**). Univariate and multivariate Cox regression analyses were applied to test the significance of the impact of TMErisk in terms of the overall survival of HGSOC patients. The TMErisk score was an independent prognostic biomarker for evaluating patient survival in various cohorts (**Figures 2C**, **S8A**). Meanwhile, the time-dependent area under the curve (AUC) suggests that the TMErisk score is a prognostic biomarker for predicting survival of HGSOC patients in the TCGA and GEO datasets (**Figure 2D**). All these results suggested that the TMErisk score had stable as well as robust performance in diverse independent cohorts. The C-index of the TMErisk score and other clinical variables in HGSOC patients were calculated, and the TMErisk score presented significantly greater accuracy than other variables (**Figures 2E**, **S8B**). To further evaluate the predictive performance of the TMErisk score in HGSOC patients, we compared our TMErisk signature with other published signatures (**Table S2**). Due to the differences in platforms, the gene expression (mRNA level) signatures that can be detected in the seven cohorts mentioned above were taken into account. A univariate Cox regression analysis and the C-index of each signature were performed. Generally, the predictive performance of the TMErisk signature was much better than that of other signatures (**Figures 2F, G**).

### Genomic status of different TMErisk groups

**Figure 3:**
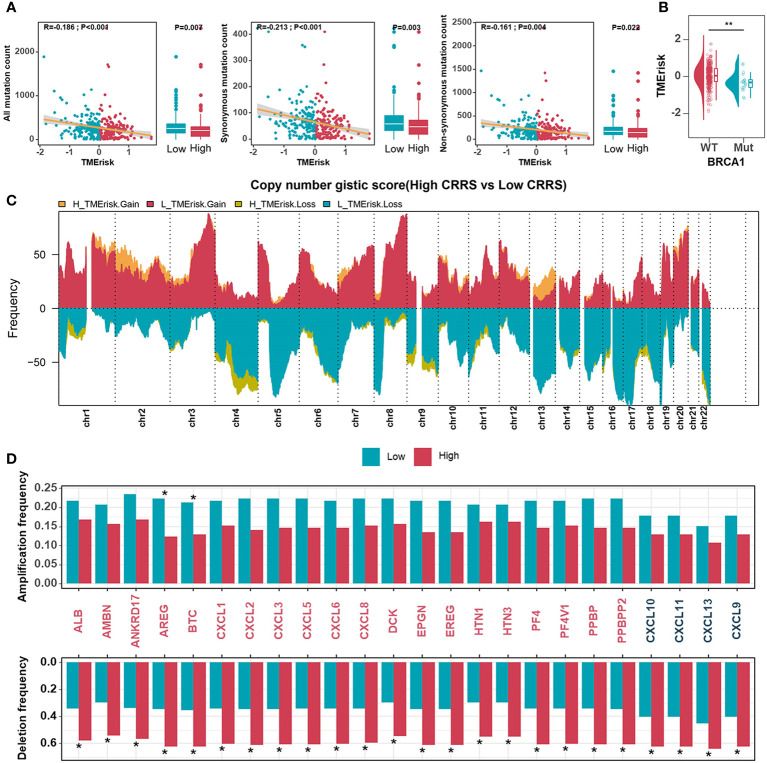
Genomic states of different TMErisk groups in HGSOC. **(A)** Boxplots comparing all mutation counts (left), synonymous mutation counts (middle), and non-synonymous mutation counts (right) between low- and high-TMErisk groups, and the correlation between mutation count and the TMErisk score in TCGA-OV cohort. **(B)** Distribution of TMErisk scores in the BRCA1 mutant and wild-type groups. **(C)** Gains and losses in copy numbers in groups with low and high TMErisk. **(D)** Copy number variations at chromosomal bands 4q13.3 and 4q21.1 between low- and high-TMErisk groups. *P < 0.05; **P < 0.01.

Somatic mutations caused by genome instability result in an abundance of neoantigens, which were thought to influence TME and contribute to effective immunotherapy. To characterize the genomic states of different TMErisk groups in the TCGA-OV database, the somatic mutation frequency was first analyzed. We identified a negative correlation between the TMErisk score and somatic mutation count, suggesting the low-TMErisk group had more somatic mutations, including synonymous and non-synonymous mutations (**Figure 3A**). The top 20 genes with the highest mutation rates in the two TMErisk groups were then identified, but there was no significant difference in mutation rates between groups (**Figure S9A**). Moreover, using Fisher’s exact test, distinct mutant genes were identified between the low- and high-TMErisk groups at a *P <* 0.05 significance level (**Figure S9B**). In the low-TMErisk group, the genes with the highest mutation rates were SETDB1, BRCA1, LRP4, XIRP1, and TOMM70A (**Figure S9B**). Preliminary evidence suggests that BRCA1/2 mutated tumors tend to contain more neoantigens and greater lymphocyte infiltration compared to non-BRCA1/2 tumors (39, 40). Here, we investigated the association between TMErisk score and BRCA1 mutation and discovered that BRCA1 mutation samples had lower TMErisk scores (**Figure 3B**). Different tumor types show a variety of copy number variations (CNVs), of which serous HGSOC has a wide and diverse alterations (5, 41). To further understand the relationship between genomic variation and TMErisk score, we analyzed and screened the CNVs in the different TMErisk groups of each group. For example, in the high TMErisk group, the genes on chromosomes 1, 2, and 13 tended to have amplified copy number, whereas on chromosomes 4 and 9, genes were likely to be deletions (**Figure 3C**). CXC chemokines and receptors are momentous for attracting immune cells from the circulatory system to inflammation or tumor sites (42). According to a recent study, a copy number deletion of chromosome arm 4q was found in an immune-cold type of HGSOC, which tended to be associated with immunosuppression (40). In detail, genes in chromosomal bands 4q13.3 (including CXCL1–3, CXCL5–6, and CXCL8) and 4q21.1 (including CXCL9/10/11, and CXCL13) were widely deleted in the high TMErisk group compared to the low TMErisk group (**Figure 3D**).

### The TMErisk score was associated with immune-related pathways

**Figure 4:**
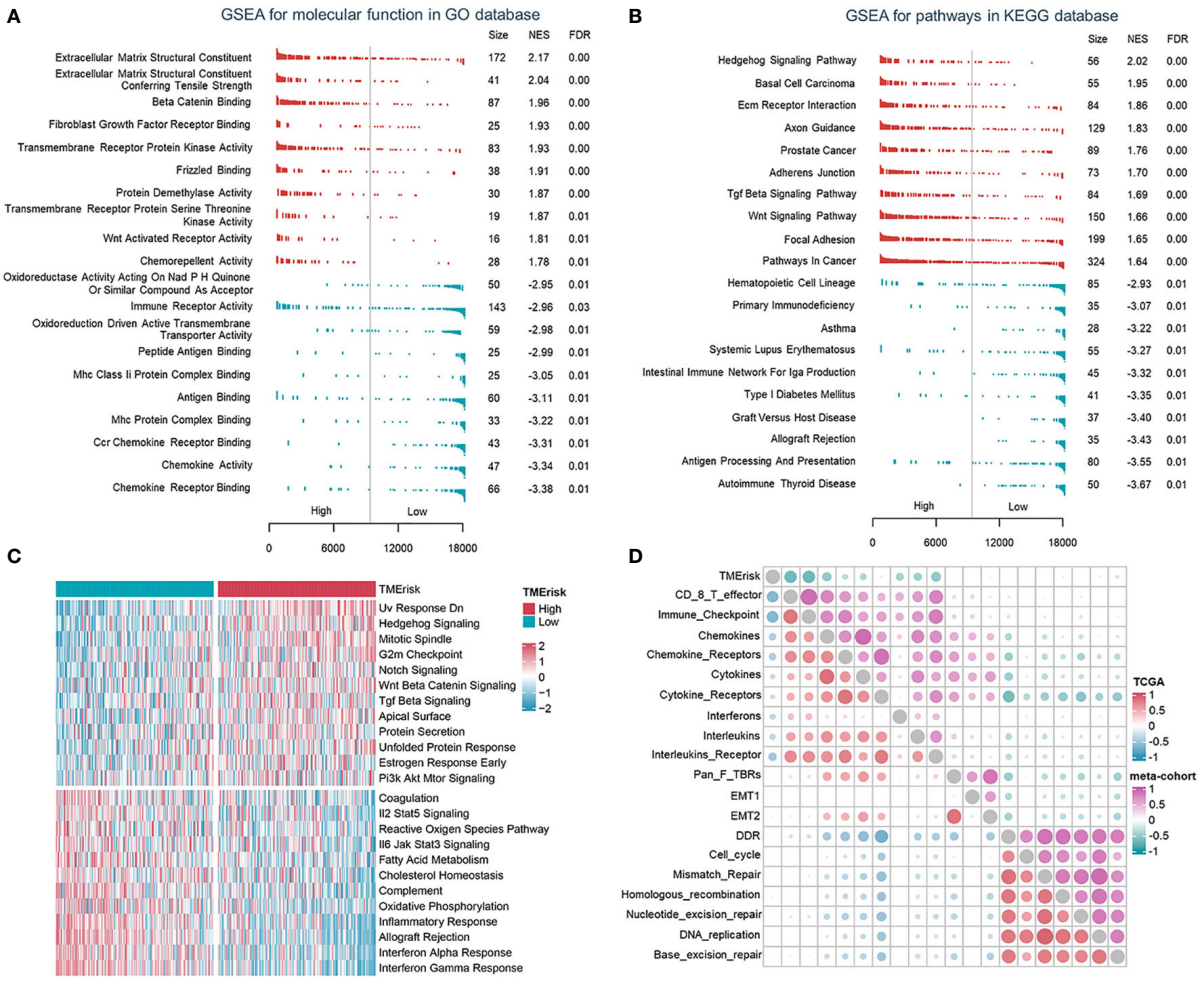
The TMErisk score was associated with immune-related pathways in HGSOC. **(A, B)** Analysis of GO molecular function **(A)** and KEGG pathway gene sets **(B)** in the low- and high-TMErisk groups. **(C)** Analysis of hallmark gene sets in the low- and high-TMErisk groups. **(D)** The correlations between TMErisk score and immune-related signatures.

To describe the biological characteristics of tumors under the TMErisk classification system, GSEA was performed with annotations of the GO and KEGG gene sets. The top 10 enriched pathways according to the normalized enriched score (NES) for each TMErisk group were displayed. ECM receptor interaction, Tgf-ß signaling, Wnt, focal adhesion, and mesenchymal cell proliferation signaling were enriched in the high TMErisk group. While gene sets associated with chemokines, chemokine receptors, antigen processing and presentation, and immunological response were enriched in the low TMErisk group (**Figures 4A, B**, **S10A**). When comparing the high- and low-TMErisk groups, GSVA enrichment analysis was also conducted. The low TMErisk group was markedly enriched in immune response-related pathways, and the high TMErisk group was enriched in pathways associated with carcinogenic activation pathways (**Figure 4C**). Moreover, the TMErisk score was adversely linked with the vast majority of immune-related signature scores (**Figure 4D**). We concluded, based on these findings, that the TMErisk scoring system effectively discriminated between distinct HGSOC tumor microenvironments.

### Immune landscapes of different TMErisk groups

**Figure 5:**
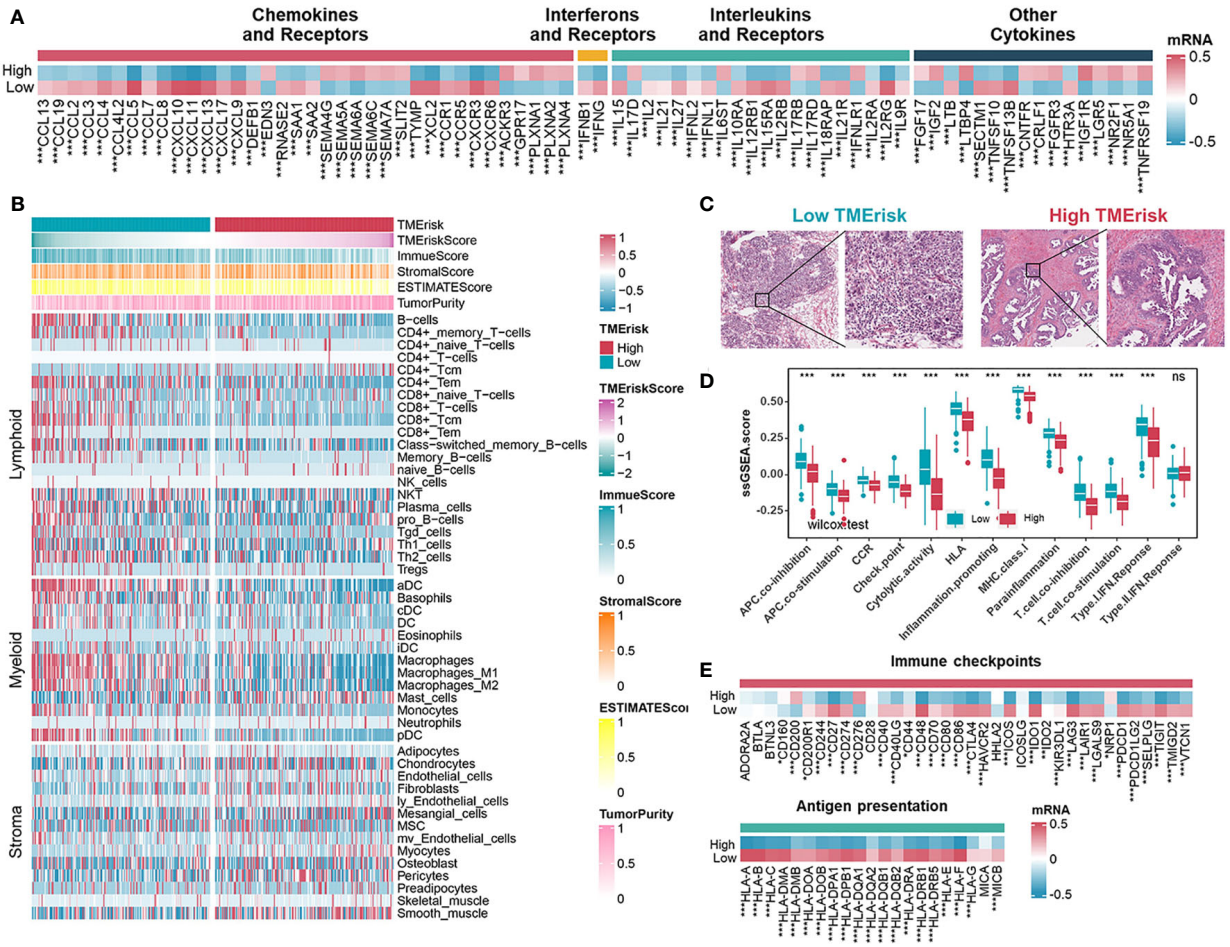
Immune landscape of different TMErisk groups in HGSOC. **(A)** Expression of chemokines, interferons, interleukins, and other cytokines in low- and high-TMErisk groups in TCGA-OV cohort. **(B)** Heatmap showing the infiltration of immune and stromal cells between low- and high-TMErisk groups in TCGA-OV cohort. **(C)** CDSA images of representative HE-stained samples of HGSOC from TCGA in low- and high-TMErisk groups. **(D, E)** Differences in immune-related functions **(D)**, immune checkpoints and HLA gene expression **(E)** between low- and high-TMErisk groups. ^ns^*P* > 0.05; *P < 0.05; **P < 0.01; ***P < 0.001

To depict the specific characteristics of TMErisk in the immune landscape of HGSOC samples, the differences in the chemokines, interleukins, and interferons between the low- and high-TMErisk groups were first compared. It has been previously shown that the CXCL9/CXCL10-CXCR3 axis is able to dictate the chemoattraction of gamma-delta T-cells, activated Th1 cells, natural killer cells, macrophages, and dendritic cells towards tumors (43, 44). The majority of chemokines/interleukins were expressed at higher levels in the low-TMErisk group compared to the high-TMErisk group, especially CXCL9/10/11 and CXCL13 located on chromosome band 4q21.1 (**Figure 5A**). Further investigation focusing on the immune components in TME between the two groups revealed that the low-TMErisk group had many signatures representing lymphoid and myeloid cells but few signatures representing stromal cells (**Figure 5B**). To ensure that the results were not biased by the analytical algorithm, the relationship between the TMErisk score and CD8+ T cells was further verified by multiple algorithms (**Figure S11A**). In the Cancer Digital Slide Archive (CDSA) database (45), we confirmed that there was more infiltration of immune cells in the tumor nests of low-TMErisk groups but less infiltration of immune cells in the tumor tissue of high-TMErisk groups (**Figure 5C**). Next, we explored associations between the TMErisk score and immune-related functions (46). The low-TMErisk group was enriched in immune activation signatures (**Figure 5D**). Meanwhile, the TMErisk score was negatively correlated with the critical steps of cancer–immunity cycle, including the release of cancer cell antigens (Step 1), priming and activation (Step 3), trafficking of immune cells to tumors (Step 4), infiltration of immune cells into tumors (Step 5), and killing of cancer cells (Step 7) (**Figure S11B**). In line with the characteristics of infiltrated immune cell and immune signatures, many immune checkpoint genes and HLA family genes were generally upregulated in low TMErisk groups indicating a tumor immune microenvironment with more neoantigens and potential effective immunotherapy (**Figure 5E**).

Besides, we further evaluated the correlation between 16 immune-related genes and immune cells, immune checkpoint genes, as well as HLA family genes. CD38 and CXCL13 showed mostly positive correlations with lymphoid and myeloid cells, immune checkpoints, and HLA genes, while others, especially SNRPE, exhibited the opposite results (**Figures S12A, B**). To investigate the roles of CD38, CXCL13, and SNRPE in the TME of HGSOC, we detected relationships between the three genes with the CD8 T effector signature (CD8A, GZMA, GZMB, IFNG, CXCL9, CXCL10, PRF1, and TBX21) and immune checkpoint signature (CD274, PDCD1LG2, CTLA4, PDCD1, LAG3, and HAVCR2) in eight cohorts. The results showed that CD8 T effector signatures and immune checkpoint signatures correlated positively with CD38 and CXCL13 but negatively with SNRPE (**Figures S12C–E**). Prior research has demonstrated that CD38 is expressed in activated T, B, and natural killer (NK) cells (47). Meanwhile, CXCL13 is typically expressed in secondary lymphoid organs by follicular dendritic cells, macrophages, and fibroblasts, and the presence of CXCL13-positive T cells has been associated with increased sensitivity to anti-PD-L1 therapy (48, 49). In this study, we sought to confirm the relationship between SNRPE and the CD8 T effector signature and immune checkpoint signature in ovarian cancer tissues. Our qRT-PCR analysis revealed a negative correlation between SNRPE expression and the expression of CD274 and CD8A in ovarian cancer tissues (**Figure S12F**).

### Predictive value of the TMErisk score in immunotherapy and chemotherapy

**Figure 6:**
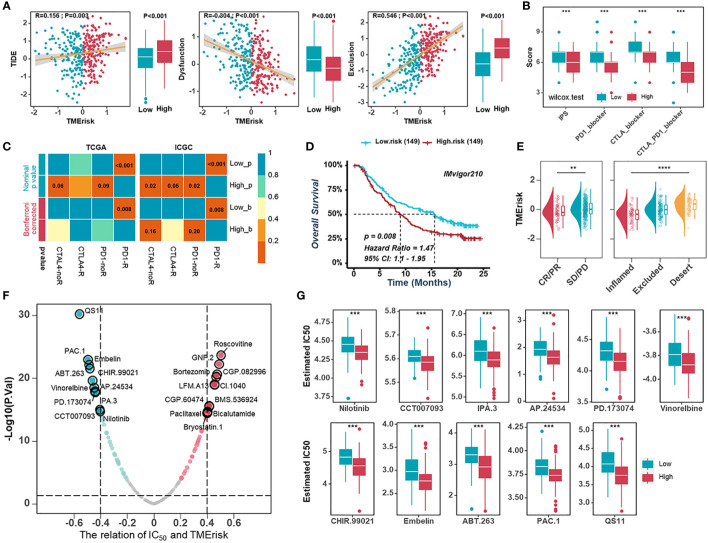
Predictive value of the TMErisk score in immunotherapy and chemotherapy. **(A)** The correlations between the TMErisk score with TIDE score (left), dysfunction score (middle), and exclusion score (right). **(B)** The correlation between the TMErisk score and IPS predictor. **(C)** Submap analyses predicting the probability of immunotherapy responses (anti-PD-1 and anti-CTLA-4) in low- and high-TMErisk groups, in TCGA-OV and ICGC cohort, respectively. **(D)** Kaplan-Meier analysis estimating the overall survival of low- and high-TMErisk groups in IMvigor210 cohort. **(E)** The distribution of TMErisk scores across groups with different immune response status (left) and immune phenotypes (right). **(F)** The relation between the IC_50_ of candidate drugs and TMErisk scores. **(G)** Boxplots showing the estimated higher IC_50_ values of drugs in the low-TMErisk group. **P < 0.01; ***P < 0.001; ****P < 0.0001.

The TMErisk score was constructed by 16 TME-related genes and associated with infiltration of immune cells, the immune checkpoint signature, and immune-related pathways. Therefore, we assumed that there were differences in immunotherapy effects for HGSOC patients with different TMErisk scores. Firstly, we applied the TIDE algorithm to assess the potential clinical efficacy of immunotherapy for TCGA-OV samples. We found that the TMErisk score was negatively correlated with dysfunction (r = -0.304, *P* < 0.001) and positively correlated with TIDE scores and exclusion (TIDE: r = 0.156, *P* = 0.003; exclusion: r = 0.546, *P* < 0.001) (**Figure 6A**). The IPS was a superior predictor to identify determinants of immunogenicity and characterize the tumor immune landscape. Higher IPS scores usually represented better outcomes with ICB treatment (50, 51). The results showed that the low TMErisk group had higher IPS, PD1-blocker, CTLA-blocker, and CTLA4-PD1-blocker scores (**Figure 6B**). In addition, we applied a subclass mapping approach to assess the treatment response of immunotherapy specifically targeting CTLA-4 and PD-1 in TCGA and ICGC samples. We discovered that patients with low TMErisk exhibited promising responses to anti-PD-1 therapy, while patients with high TMErisk showed no responses to anti-PD-1 therapy (**Figure 6C**). In the IMvigor210 cohort, we investigated whether TMErisk could predict patient response to the ICB therapy in an independent immunotherapy cohort. As expected, the patients with a higher TMErisk score were less likely to benefit from immune checkpoint therapy and had a worse prognosis than those with a lower TMErisk score (**Figures 6D, E**).

To study further the treatment methods for the various TMErisk groups, the pRRophetic software was used to predict the medication response of each sample. We investigated correlations between the TMErisk score and the IC_50_ of drug candidates in the GDSC database. Using Spearman correlation analysis, we discovered that the IC_50_ of eleven candidates was positively correlated with the TMErisk score, while the IC_50_ of eleven other drugs was negatively correlated (**Figure 6F**). The predicted IC_50_ of these medicines differed significantly across the two TMErisk groups (**Figures 6G**, **S13A**). Paclitaxel was considered the first-line drug for HGSOC treatment among these drugs, and patients in the low-TMErisk group may be more sensitive to Paclitaxel (**Figure S13A**).

## Discussion

HGSOC tumors are comprised of multiple populations of various tumor, immune, and stromal cells that are inherently heterogeneous and could develop different phenotypes. Pathbreaking research by Tothill et al. (4) identified six subtypes of HGSOC through optimal clustering of array data. Significantly, patients from the high stromal response subtype (C1) had the poorest survival. C2 and C4 subtypes with more abundant CD3+ cells and lower expression of stromal genes had better survival than C1. They also identified a high-grade serous subtype with a mesenchymal expression pattern, characterized by highly expressed N-cadherin and P-cadherin and low expression of differentiation markers, with relatively reduced OS compared with C2 and C4 subtypes. Similarly, TCGA project delineated four HGSOC transcriptional subtypes, including proliferative, mesenchymal, differentiated, and immunoreactive subtypes, generally suggesting that, in addition to molecular subtypes of tumor cells, heterogeneity in proportion and anatomical location of non-tumor cells also leads to different phenotypes (5). For example, high expression of HOX genes and markers suggestive of increased stromal components characterized the mesenchymal subtype. T-cell chemokine ligands and receptors characterize the immunoreactive subtype. However, some research casts doubt on these models due to independent validation efforts that failed to identify subtypes or only two or three reproducible subtypes (6, 52). A large proportion of models exhibited lower accuracy in other new datasets than in the validation sets used in their own papers. And the robustness across studies and clinical relevance of these subtypes require improvement to be of value (7). Significant effort is needed to translate these subtypes into clinical practice.

Heterogeneity and diversity of cell composition pose a major challenge to determining the immune landscape and effective immunotherapy in HGSOC. Given the previously reported prognostic significance of intertumoral T cells within HGSOC (12, 53), CD8+ TILs are undoubtedly a key factor in certain histotypes of HGSOC and need to be studied additionally for immunotherapy (13). The immunoreactive subtype from TCGA is so named because these tumors display prominent T cell infiltration (5). Additionally, stromal cells are a significant population of cells that also influence immunological state and subtyping. Cancer-associated fibroblasts (mCAF) in matrix expressing vimentin, SMA, COL3A, COL10, and MMP11 were predominant in HGSOC tumors and were capable of inducing EMT characteristics in HGSOC cells (18). Meanwhile, an elevated stromal response and its relevant gene expression signature are significant prognostic indicators within HGSOC (4). Therefore, it is desirable to consider the immune cells as well as stromal cells in a coordinated way for elucidating the TME of HGSOC.

Out of regard for consistent results across independent cohorts and clinical feasibility, we extensively collect multiple HGSOC datasets. The consensus cluster analyses were performed in seven independent cohorts and an integrated meta-cohort to identify tumor-immune-stroma phenotypes. Explicitly, two clusters could achieve the best clustering efficacy in the meta-cohort, and consistent clustering results were obtained in all independent cohorts. When establishing the TMErisk scoring system, we integrated ten independent machine learning algorithms to acquire the TMErisk with stable performance and high accuracy in different cohorts. 108 combinations of prediction models were fitted out in the TCGA-OV cohort, and the average C-index of each model in the other seven validation cohorts was further calculated. To confirm the robust and stable performance of the TMErisk in multiple independent cohorts, we compared our TMErisk signature with other published signatures. Univariate Cox regression analysis and the C-index of each signature revealed that the predictive performance of the TMErisk signature was much better than that of other signatures.

The close association of somatically mutated genes with specific tumor subtypes or immunological phenotypes led to the discovery of molecules or antibodies specific to these cancer targets (54). To identify possible targets influencing TME and contributing to the efficacy of immunotherapy for patients with different TMErisk scores, we examined the frequency of somatic mutations and copy number variations. Preliminary studies suggested HGSOC patients with BRCA1 mutations demonstrated higher CD8+ TILs, and neoantigen load might explain higher CD8+ TILs (39, 40), which is consistent with patients with low TMErisk scores exhibiting more neoantigens and an increased number of tumor-infiltrating lymphocytes. It has been suggested that copy number variations may contribute more than somatic mutations to the process of tumorigenesis (55). T-cell chemokine ligands CXCL11 and CXCL10, and their receptor CXCR3, characterized the immunoreactive subtype of TCGA, which is consistent with that in the low-TMErisk group in our study. CNV analysis reminded us of the mechanism underlying the abnormal expression of chemokines and relevant receptors and revealed that the 4q21.1 region (including CXCL9/10/11 and CXCL13) was widely deleted in the high TMErisk group compared to the low TMErisk group, probably explaining the prominent T cell infiltration in the low TMErisk group.

To develop a patient-specific treatment based on the phenotyping of HGSOC tumors, we evaluated the effectiveness of the TMErisk score in guiding immunotherapy and chemotherapy. Using TIDE, IPS and subclass mapping to measure the immune response, HGSOC patients with a high TMErisk score were not only less likely to respond to ICB treatment but also more susceptible to immunological escape. In addition, a patient with a low TMErisk score and a positive response to ICBs were observed in anti-PD-1 immunotherapy cohorts. Moreover, we recognize some medications with considerably distinct IC_50_ estimates between two TMErisk groups. Among them, Paclitaxel was regarded as the first-line treatment for HGSOC, and individuals with a low TMErisk were more likely to be sensitive to the medicine.

The current study has several limitations that warrant discussion. Firstly, the results were derived from an online database, and all samples were retrospective, necessitating larger clinical trials, particularly prospective trials, to validate the findings. Secondly, we screened many genes that are associated with the immune microenvironment of ovarian cancer, and further *in vitro* and *in vivo* experiments are necessary to confirm the function of these genes. Furthermore, owing to the lack of immunotherapy information for ovarian cancer, the study only confirmed the association between TMErisk and immunotherapy response through website predictions and the analysis of the IMvigor210 cohort. Therefore, a new ovarian cancer cohort is required for further investigation.

In conclusion, our study not only depicts the diversity of cell components in the TME of HGSOC, but also highlights the contributions of the cross-talk within those components in shaping the biology of the TME, which eventually influences the patients’ response to immunotherapies. To address the robustness across studies and clinical relevance of subtyping when designing a prognostic scoring system for HGSOC patients, we have performed a machine learning-based procedure to guide the identification of the TMErisk score, achieving high accuracy and stability performance in different independent cohorts. Significantly, the predictive performance of the TMErisk signature was much better than other published signatures. Finally, our findings assist to identify potential targets and provide novel therapeutic strategies for addressing tumor immunosuppression and enhancing the response to cancer therapy.

## Data availability statement

The datasets presented in this study can be found in online repositories. The names of the repository/repositories and accession number(s) can be found within the article/**Supplementary Materials**.

## Author contributions

YL and XF conceived the work and analyzed the data. RT and QW prepared the figures and drafted the manuscript. JL, CO, XH and XF edited and revised the manuscript. All authors contributed to the article and approved the submitted version.

## References

[1] Siegel RL Miller KD Fuchs HE Jemal A. Cancer statistics, 2022. CA Cancer J Clin (2022) 72(1):7–33. doi: 10.3322/caac.21708 35020204

[2] Vaughan S Coward JI Bast RC Jr. Berchuck A Berek JS Brenton JD. Rethinking ovarian cancer: Recommendations for improving outcomes. Nat Rev Cancer (2011) 11(10):719–25. doi: 10.1038/nrc3144 PMC3380637 21941283

[3] Seidman JD Horkayne-Szakaly I Haiba M Boice CR Kurman RJ Ronnett BM. The histologic type and stage distribution of ovarian carcinomas of surface epithelial origin. Int J Gynecol Pathol (2004) 23(1):41–4. doi: 10.1097/01.pgp.0000101080.35393.16 14668549

[4] Tothill RW Tinker AV George J Brown R Fox SB Lade S. Novel molecular subtypes of serous and endometrioid ovarian cancer linked to clinical outcome. Clin Cancer Res (2008) 14(16):5198–208. doi: 10.1158/1078-0432.CCR-08-0196 18698038

[5] Cancer Genome Atlas Research N. Integrated genomic analyses of ovarian carcinoma. Nature (2011) 474(7353):609–15. doi: 10.1038/nature10166 PMC3163504 21720365

[6] Chen GM Kannan L Geistlinger L Kofia V Safikhani Z Gendoo DMA. Consensus on molecular subtypes of high-grade serous ovarian carcinoma. Clin Cancer Res (2018) 24(20):5037–47. doi: 10.1158/1078-0432.CCR-18-0784 PMC6207081 30084834

[7] Waldron L Haibe-Kains B Culhane AC Riester M Ding J Wang XV. Comparative meta-analysis of prognostic gene signatures for late-stage ovarian cancer. J Natl Cancer Inst (2014) 106(5). doi: 10.1093/jnci/dju049 PMC4580554 24700801

[8] Brahmer J Reckamp KL Baas P Crino L Eberhardt WE Poddubskaya E. Nivolumab versus docetaxel in advanced squamous-cell non-Small-Cell lung cancer. N Engl J Med (2015) 373(2):123–35. doi: 10.1056/NEJMoa1504627 PMC4681400 26028407

[9] Wolchok JD Chiarion-Sileni V Gonzalez R Rutkowski P Grob JJ Cowey CL. Overall survival with combined nivolumab and ipilimumab in advanced melanoma. N Engl J Med (2017) 377(14):1345–56. doi: 10.1056/NEJMoa1709684 PMC5706778 28889792

[10] Matulonis UA Shapira-Frommer R Santin AD Lisyanskaya AS Pignata S Vergote I. Antitumor activity and safety of pembrolizumab in patients with advanced recurrent ovarian cancer: Results from the phase ii keynote-100 study. Ann Oncol (2019) 30(7):1080–7. doi: 10.1093/annonc/mdz135 31046082

[11] Hamanishi J Mandai M Konishi I. Immune checkpoint inhibition in ovarian cancer. Int Immunol (2016) 28(7):339–48. doi: 10.1093/intimm/dxw020 27055470

[12] Zhang L Conejo-Garcia JR Katsaros D Gimotty PA Massobrio M Regnani G. Intratumoral T cells, recurrence, and survival in epithelial ovarian cancer. N Engl J Med (2003) 348(3):203–13. doi: 10.1056/NEJMoa020177 12529460

[13] Ovarian Tumor Tissue Analysis C Goode EL Block MS Kalli KR Vierkant RA Chen W. Dose-response association of Cd8+ tumor-infiltrating lymphocytes and survival time in high-grade serous ovarian cancer. JAMA Oncol (2017) 3(12):e173290. doi: 10.1001/jamaoncol.2017.3290 29049607 PMC5744673

[14] Tanaka Y Kobayashi H Suzuki M Hirashima Y Kanayama N Terao T. Genetic downregulation of pregnancy-associated plasma protein-a (Papp-a) by bikunin reduces igf-I-Dependent akt and Erk1/2 activation and subsequently reduces ovarian cancer cell growth, invasion and metastasis. Int J Cancer (2004) 109(3):336–47. doi: 10.1002/ijc.11700 14961570

[15] Boldt HB Conover CA. Overexpression of pregnancy-associated plasma protein-a in ovarian cancer cells promotes tumor growth in vivo. Endocrinology (2011) 152(4):1470–8. doi: 10.1210/en.2010-1095 21303951

[16] Hornburg M Desbois M Lu S Guan Y Lo AA Kaufman S. Single-cell dissection of cellular components and interactions shaping the tumor immune phenotypes in ovarian cancer. Cancer Cell (2021) 39(7):928–44 e6. doi: 10.1016/j.ccell.2021.04.004 33961783

[17] Olbrecht S Busschaert P Qian J Vanderstichele A Loverix L Van Gorp T. High-grade serous tubo-ovarian cancer refined with single-cell rna sequencing: Specific cell subtypes influence survival and determine molecular subtype classification. Genome Med (2021) 13(1):111. doi: 10.1186/s13073-021-00922-x 34238352 PMC8268616

[18] Xu J Fang Y Chen K Li S Tang S Ren Y. Single-cell rna sequencing reveals the tissue architecture in human high-grade serous ovarian cancer. Clin Cancer Res (2022) 28(16):3590–602. doi: 10.1158/1078-0432.CCR-22-0296 PMC9662915 35675036

[19] Li Y Tian R Liu J Li J Tan H Wu Q. Deciphering the immune landscape dominated by cancer-associated fibroblasts to investigate their potential in indicating prognosis and guiding therapeutic regimens in high grade serous ovarian carcinoma. Front Immunol (2022) 13:940801. doi: 10.3389/fimmu.2022.940801 36119108 PMC9478207

[20] Liu J Wang Y Yuan S Wei J Bai J. Construction of an immune cell infiltration score to evaluate the prognosis and therapeutic efficacy of ovarian cancer patients. Front Immunol (2021) 12:751594. doi: 10.3389/fimmu.2021.751594 34745124 PMC8564196

[21] Li Y Gan Y Liu J Li J Zhou Z Tian R. Downregulation of Meis1 mediated by Elfn1-As1/Ezh2/Dnmt3a axis promotes tumorigenesis and oxaliplatin resistance in colorectal cancer. Signal Transduct Target Ther (2022) 7(1):87. doi: 10.1038/s41392-022-00902-6 35351858 PMC8964798

[22] Li Y Liu J Xiao Q Tian R Zhou Z Gan Y. En2 as an oncogene promotes tumor progression *Via* regulating Ccl20 in colorectal cancer. Cell Death Dis (2020) 11(7):604. doi: 10.1038/s41419-020-02804-3 32732864 PMC7393501

[23] Charoentong P Finotello F Angelova M Mayer C Efremova M Rieder D. Pan-cancer immunogenomic analyses reveal genotype-immunophenotype relationships and predictors of response to checkpoint blockade. Cell Rep (2017) 18(1):248–62. doi: 10.1016/j.celrep.2016.12.019 28052254

[24] Zeng D Ye Z Shen R Yu G Wu J Xiong Y. Iobr: Multi-omics immuno-oncology biological research to decode tumor microenvironment and signatures. Front Immunol (2021) 12:687975. doi: 10.3389/fimmu.2021.687975 34276676 PMC8283787

[25] Newman AM Liu CL Green MR Gentles AJ Feng W Xu Y. Robust enumeration of cell subsets from tissue expression profiles. Nat Methods (2015) 12(5):453–7. doi: 10.1038/nmeth.3337 PMC4739640 25822800

[26] Racle J Gfeller D. Epic: A tool to estimate the proportions of different cell types from bulk gene expression data. Methods Mol Biol (2020) 2120:233–48. doi: 10.1007/978-1-0716-0327-7_17 32124324

[27] Finotello F Mayer C Plattner C Laschober G Rieder D Hackl H. Molecular and pharmacological modulators of the tumor immune contexture revealed by deconvolution of rna-seq data. Genome Med (2019) 11(1):34. doi: 10.1186/s13073-019-0638-6 31126321 PMC6534875

[28] Aran D Hu Z Butte AJ. Xcell: Digitally portraying the tissue cellular heterogeneity landscape. Genome Biol (2017) 18(1):220. doi: 10.1186/s13059-017-1349-1 29141660 PMC5688663

[29] Becht E Giraldo NA Lacroix L Buttard B Elarouci N Petitprez F. Estimating the population abundance of tissue-infiltrating immune and stromal cell populations using gene expression. Genome Biol (2016) 17(1):218. doi: 10.1186/s13059-016-1070-5 27765066 PMC5073889

[30] Wilkerson MD Hayes DN. Consensusclusterplus: A class discovery tool with confidence assessments and item tracking. Bioinformatics (2010) 26(12):1572–3. doi: 10.1093/bioinformatics/btq170 PMC2881355 20427518

[31] Yu Z Lan J Li W Jin L Qi F Yu C. Circular rna Hsa_Circ_0002360 promotes proliferation and invasion and inhibits oxidative stress in gastric cancer by sponging mir-629-3p and regulating the Pdlim4 expression. Oxid Med Cell Longev (2022) 2022:2775433. doi: 10.1155/2022/2775433 35982735 PMC9381216

[32] Liu Z Liu L Weng S Guo C Dang Q Xu H. Machine learning-based integration develops an immune-derived lncrna signature for improving outcomes in colorectal cancer. Nat Commun (2022) 13(1):816. doi: 10.1038/s41467-022-28421-6 35145098 PMC8831564

[33] Liu Z Guo C Dang Q Wang L Liu L Weng S. Integrative analysis from multi-center studies identities a consensus machine learning-derived lncrna signature for stage Ii/Iii colorectal cancer. EBioMedicine (2022) 75:103750. doi: 10.1016/j.ebiom.2021.103750 34922323 PMC8686027

[34] Wu T Hu E Xu S Chen M Guo P Dai Z. Clusterprofiler 4.0: A universal enrichment tool for interpreting omics data. Innovation (Camb) (2021) 2(3):100141. doi: 10.1016/j.xinn.2021.100141 34557778 PMC8454663

[35] Hanzelmann S Castelo R Guinney J. Gsva: Gene set variation analysis for microarray and rna-seq data. BMC Bioinf (2013) 14:7. doi: 10.1186/1471-2105-14-7 PMC3618321 23323831

[36] Jiang P Gu S Pan D Fu J Sahu A Hu X. Signatures of T cell dysfunction and exclusion predict cancer immunotherapy response. Nat Med (2018) 24(10):1550–8. doi: 10.1038/s41591-018-0136-1 PMC6487502 30127393

[37] Hoshida Y Brunet JP Tamayo P Golub TR Mesirov JP. Subclass mapping: Identifying common subtypes in independent disease data sets. PloS One (2007) 2(11):e1195. doi: 10.1371/journal.pone.0001195 18030330 PMC2065909

[38] Lu X Jiang L Zhang L Zhu Y Hu W Wang J. Immune signature-based subtypes of cervical squamous cell carcinoma tightly associated with human papillomavirus type 16 expression, molecular features, and clinical outcome. Neoplasia (2019) 21(6):591–601. doi: 10.1016/j.neo.2019.04.003 31055200 PMC6658934

[39] Launonen IM Lyytikainen N Casado J Anttila EA Szabo A Haltia UM. Single-cell tumor-immune microenvironment of Brca1/2 mutated high-grade serous ovarian cancer. Nat Commun (2022) 13(1):835. doi: 10.1038/s41467-022-28389-3 35149709 PMC8837628

[40] Sun J Yan C Xu D Zhang Z Li K Li X. Immuno-genomic characterisation of high-grade serous ovarian cancer reveals immune evasion mechanisms and identifies an immunological subtype with a favourable prognosis and improved therapeutic efficacy. Br J Cancer (2022) 126(11):1570–80. doi: 10.1038/s41416-021-01692-4 PMC9130248 35017656

[41] Graf RP Eskander R Brueggeman L Stupack DG. Association of copy number variation signature and survival in patients with serous ovarian cancer. JAMA Netw Open (2021) 4(6):e2114162. doi: 10.1001/jamanetworkopen.2021.14162 34181012 PMC8239953

[42] Huang X Hao J Tan YQ Zhu T Pandey V Lobie PE. Cxc chemokine signaling in progression of epithelial ovarian cancer: Theranostic perspectives. Int J Mol Sci (2022) 23(5). doi: 10.3390/ijms23052642 PMC8910147 35269786

[43] Rainczuk A Rao J Gathercole J Stephens AN. The emerging role of cxc chemokines in epithelial ovarian cancer. Reproduction (2012) 144(3):303–17. doi: 10.1530/REP-12-0153 22771929

[44] Strieter RM Burdick MD Mestas J Gomperts B Keane MP Belperio JA. Cancer cxc chemokine networks and tumour angiogenesis. Eur J Cancer (2006) 42(6):768–78. doi: 10.1016/j.ejca.2006.01.006 16510280

[45] Gutman DA Cobb J Somanna D Park Y Wang F Kurc T. Cancer digital slide archive: An informatics resource to support integrated in silico analysis of tcga pathology data. J Am Med Inform Assoc (2013) 20(6):1091–8. doi: 10.1136/amiajnl-2012-001469 PMC3822112 23893318

[46] Zheng S Liang JY Tang Y Xie J Zou Y Yang A. Dissecting the role of cancer-associated fibroblast-derived biglycan as a potential therapeutic target in immunotherapy resistance: A tumor bulk and single-cell transcriptomic study. Clin Transl Med (2023) 13(2):e1189. doi: 10.1002/ctm2.1189 36772945 PMC9920016

[47] Frasca L Fedele G Deaglio S Capuano C Palazzo R Vaisitti T. Cd38 orchestrates migration, survival, and Th1 immune response of human mature dendritic cells. Blood (2006) 107(6):2392–9. doi: 10.1182/blood-2005-07-2913 16293598

[48] Beider K Voevoda-Dimenshtein V Zoabi A Rosenberg E Magen H Ostrovsky O. Cxcl13 chemokine is a novel player in multiple myeloma osteolytic microenvironment, M2 macrophage polarization, and tumor progression. J Hematol Oncol (2022) 15(1):144. doi: 10.1186/s13045-022-01366-5 36217194 PMC9549634

[49] Zou Y Ye F Kong Y Hu X Deng X Xie J. The single-cell landscape of intratumoral heterogeneity and the immunosuppressive microenvironment in liver and brain metastases of breast cancer. Adv Sci (Weinh) (2023) 10(5):e2203699. doi: 10.1002/advs.202203699 36529697 PMC9929130

[50] Huang X Zhao L Jin Y Wang Z Li T Xu H. Up-regulated misp is associated with poor prognosis and immune infiltration in pancreatic ductal adenocarcinoma. Front Oncol (2022) 12:827051. doi: 10.3389/fonc.2022.827051 35433491 PMC9005831

[51] Yang Z Yan G Zheng L Gu W Liu F Chen W. Ykt6, as a potential predictor of prognosis and immunotherapy response for oral squamous cell carcinoma, is related to cell invasion, metastasis, and Cd8+ T cell infiltration. Oncoimmunology (2021) 10(1):1938890. doi: 10.1080/2162402X.2021.1938890 34221701 PMC8224202

[52] Way GP Rudd J Wang C Hamidi H Fridley BL Konecny GE. Comprehensive cross-population analysis of high-grade serous ovarian cancer supports no more than three subtypes. G3 (Bethesda) (2016) 6(12):4097–103. doi: 10.1534/g3.116.033514 PMC5144978 27729437

[53] Verhaak RG Tamayo P Yang JY Hubbard D Zhang H Creighton CJ. Prognostically relevant gene signatures of high-grade serous ovarian carcinoma. J Clin Invest (2013) 123(1):517–25. doi: 10.1172/JCI65833 PMC3533304 23257362

[54] Hahn WC Bader JS Braun TP Califano A Clemons PA Druker BJ. An expanded universe of cancer targets. Cell (2021) 184(5):1142–55. doi: 10.1016/j.cell.2021.02.020 PMC8066437 33667368

[55] Alexandrov LB Nik-Zainal S Wedge DC Aparicio SA Behjati S Biankin AV. Signatures of mutational processes in human cancer. Nature (2013) 500(7463):415–21. doi: 10.1038/nature12477 PMC3776390 23945592

