# Supplementary figures and images for: Machine learning-based integration develops an immune-related risk model for predicting prognosis of high-grade serous ovarian cancer and providing therapeutic strategies
Source: Front Immunol. 2023 Apr 5;14:1164408. doi: 10.3389/fimmu.2023.1164408 (PMC10113544; doi:10.3389/fimmu.2023.1164408)

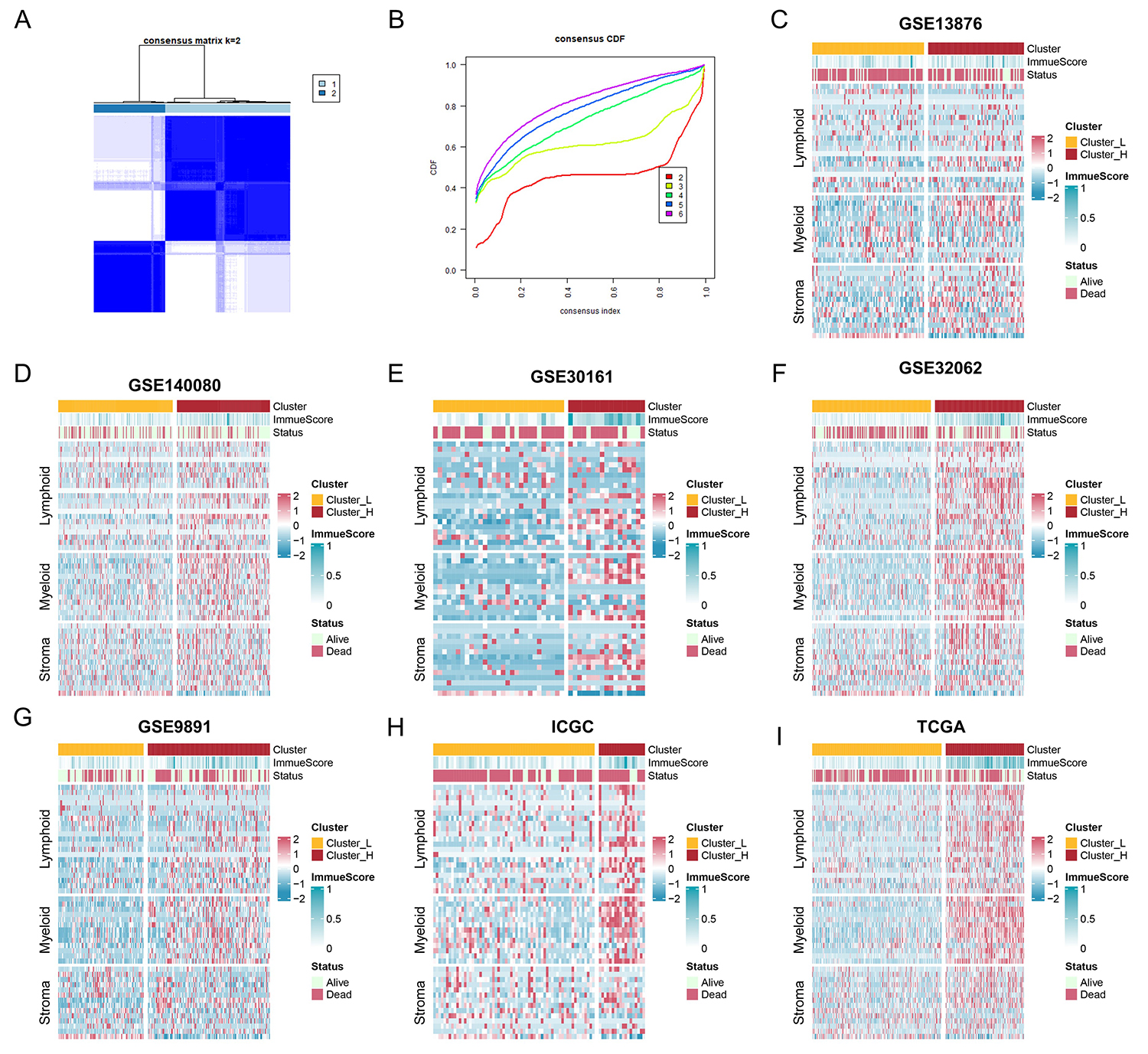

Supplement: Supplementary file 2 [file Image_1.jpeg]

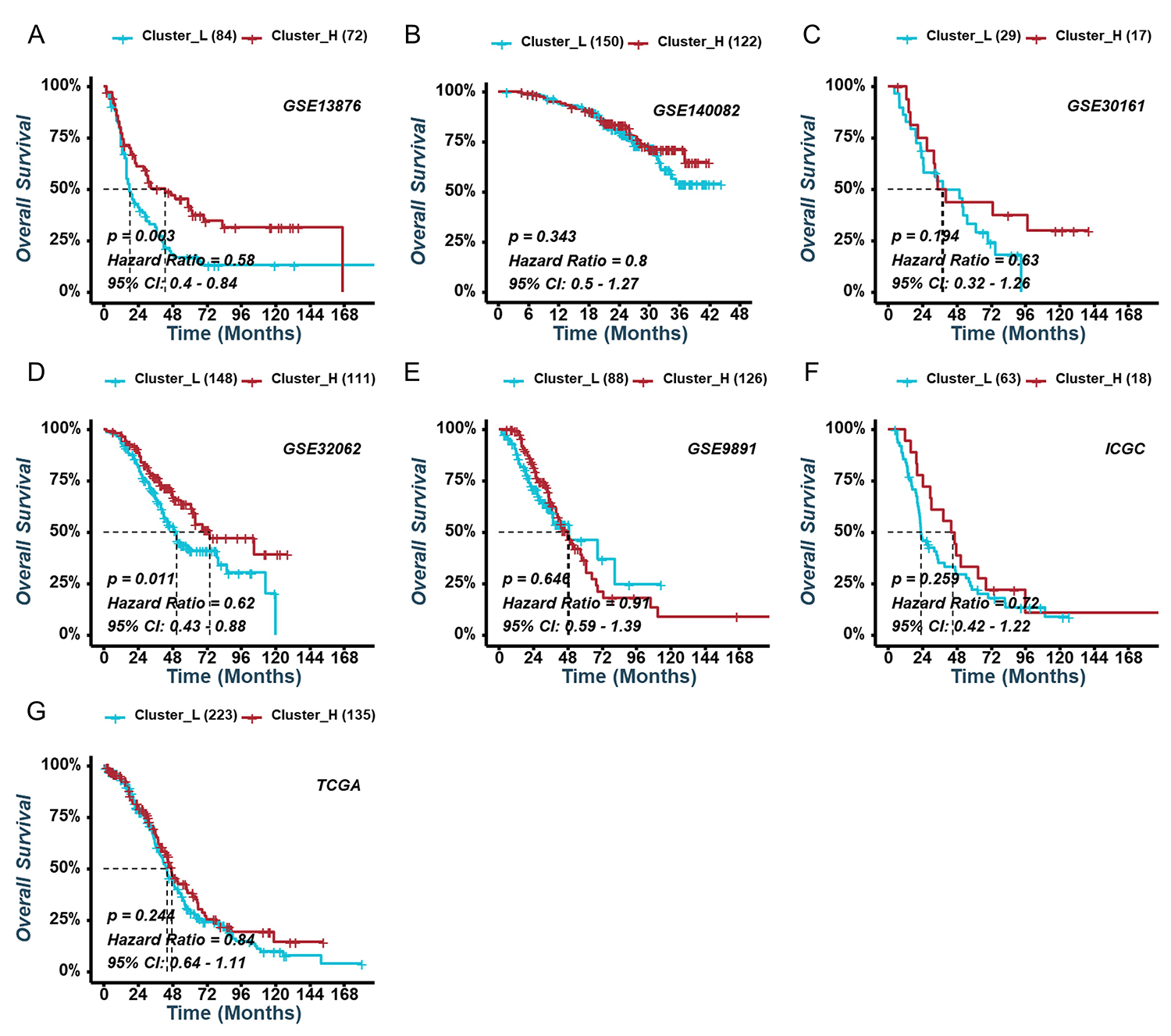

Supplement: Supplementary file 3 [file Image_2.jpeg]

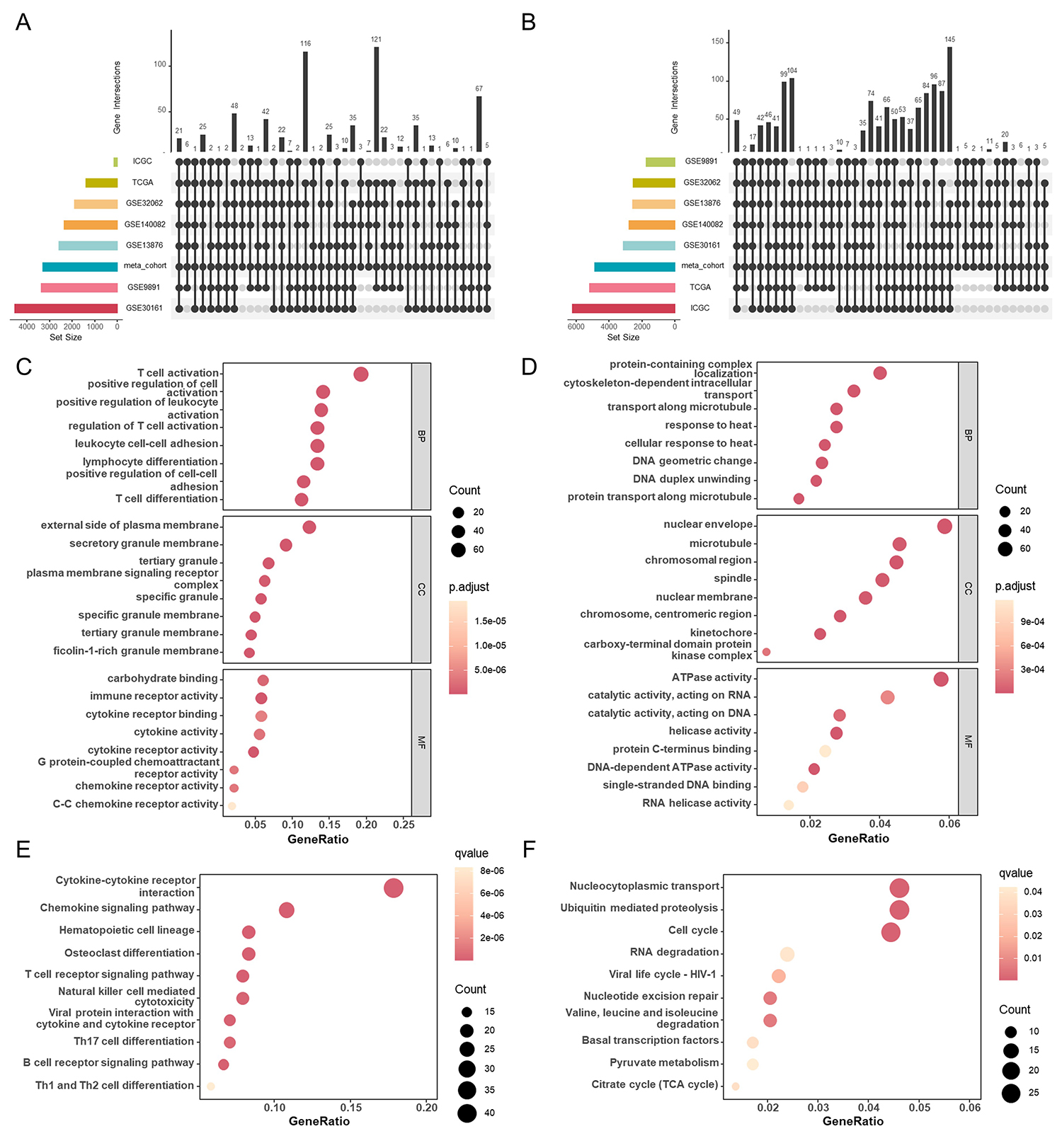

Supplement: Supplementary file 4 [file Image_3.jpeg]

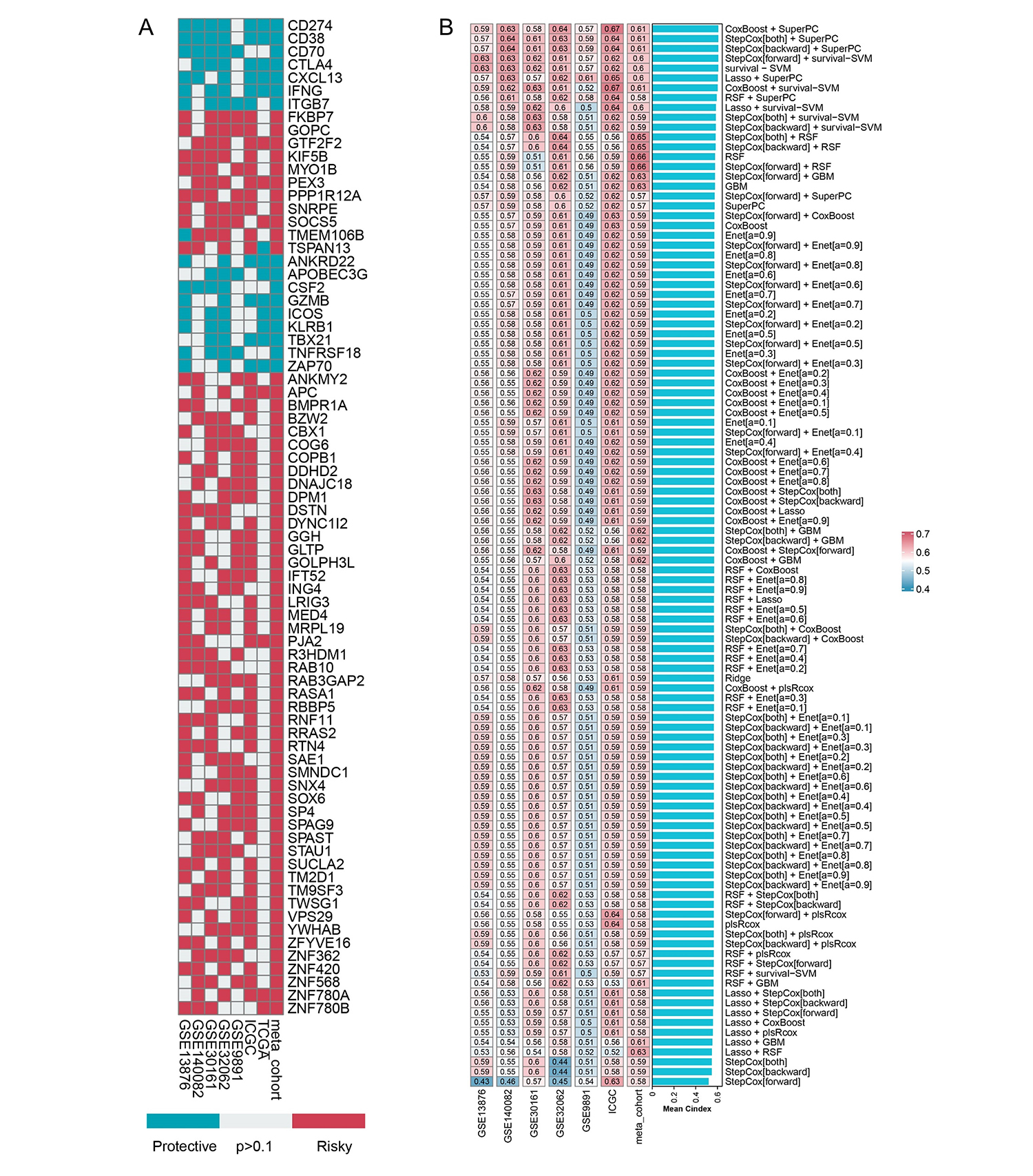

Supplement: Supplementary file 5 [file Image_4.jpeg]

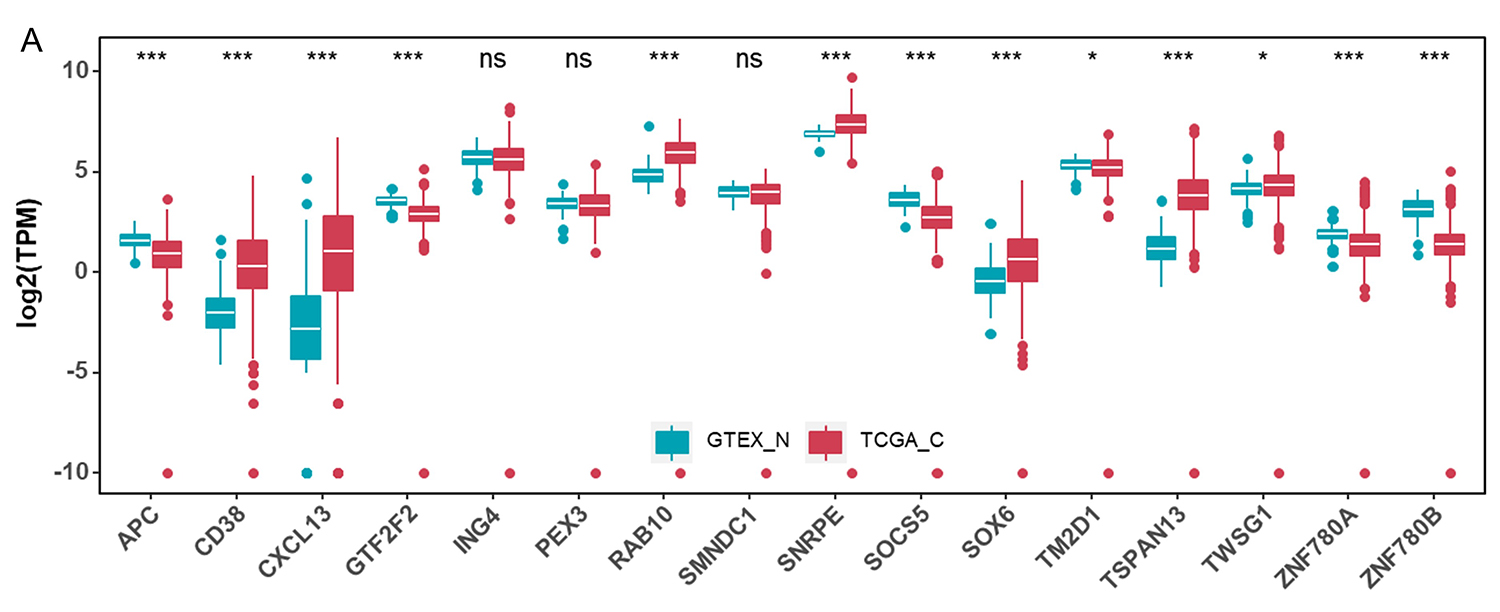

Supplement: Supplementary file 6 [file Image_5.jpeg]

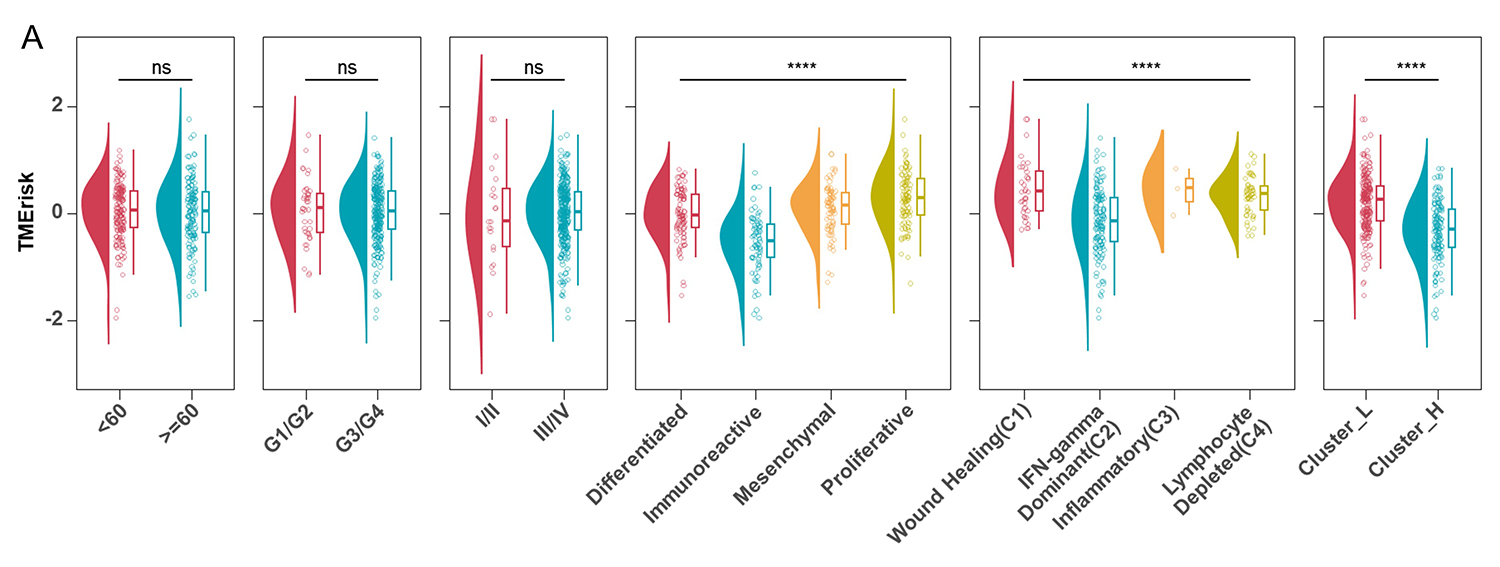

Supplement: Supplementary file 7 [file Image_6.jpeg]

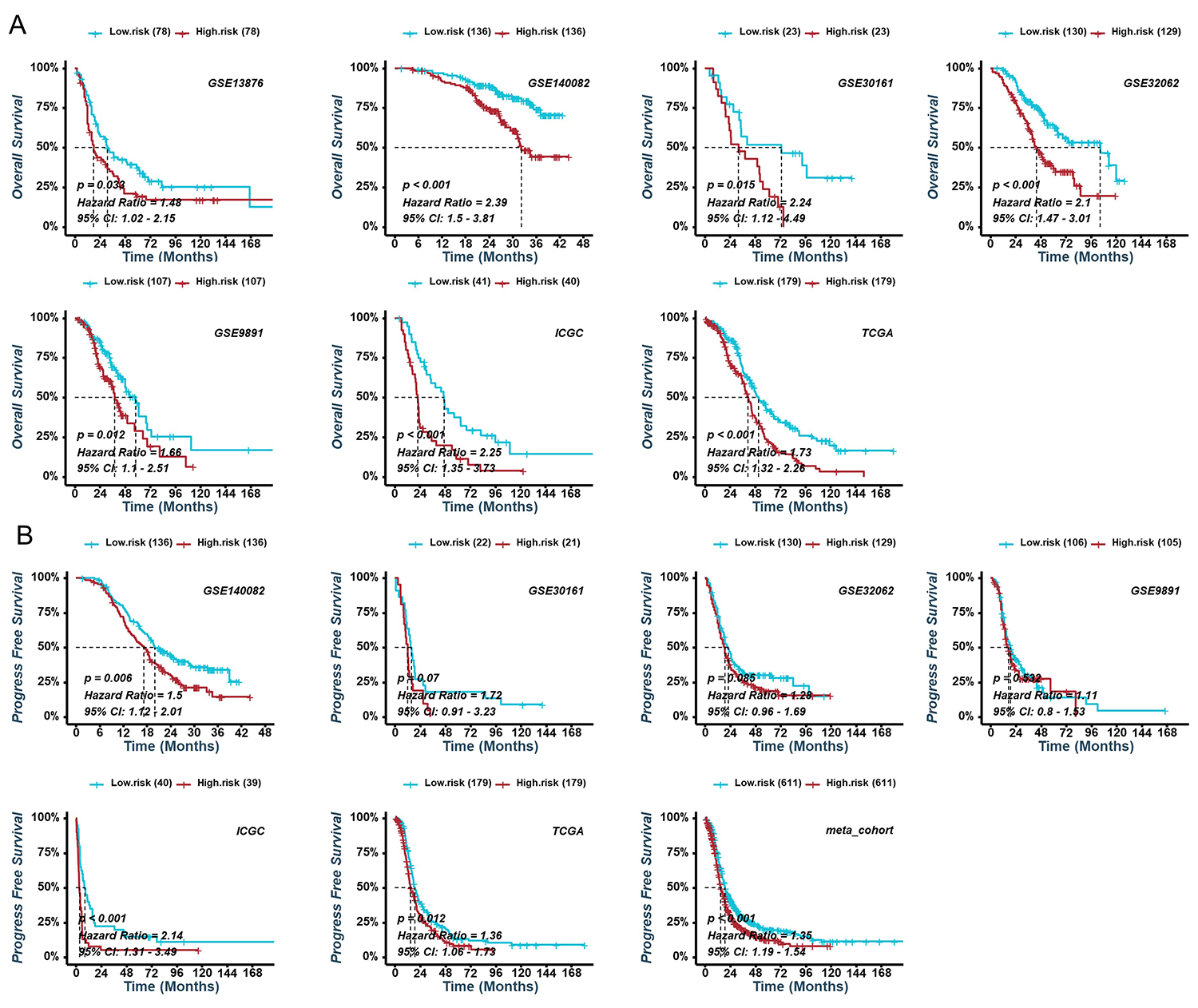

Supplement: Supplementary file 8 [file Image_7.jpeg]

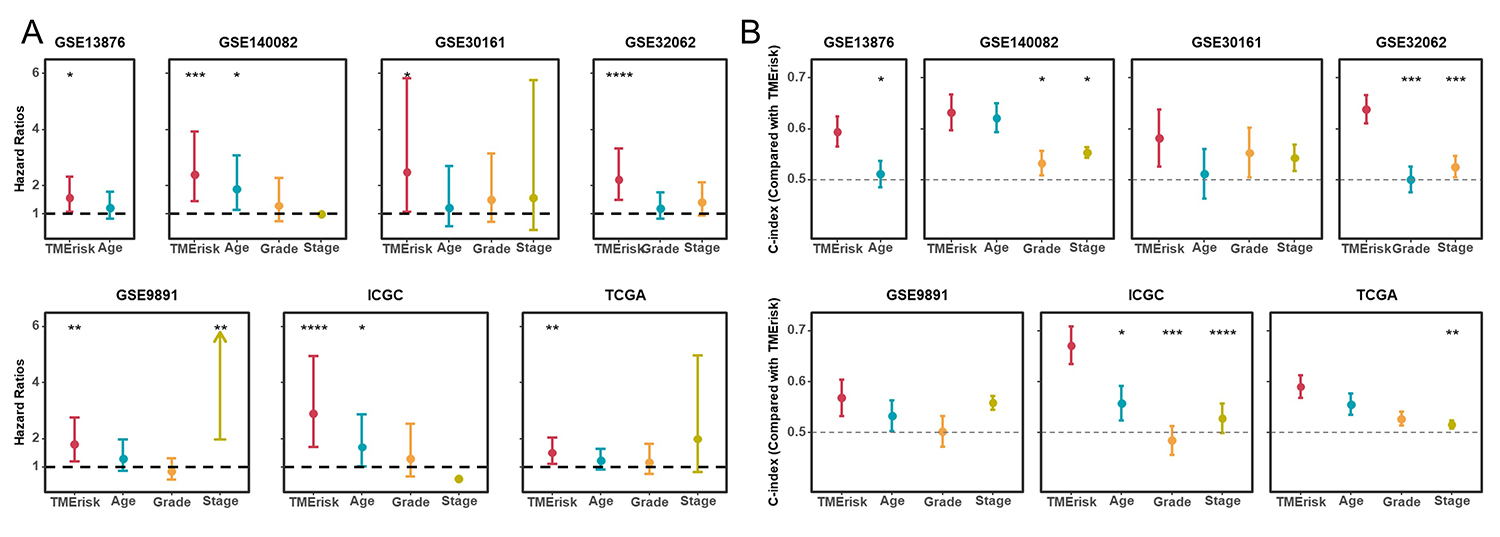

Supplement: Supplementary file 9 [file Image_8.jpeg]

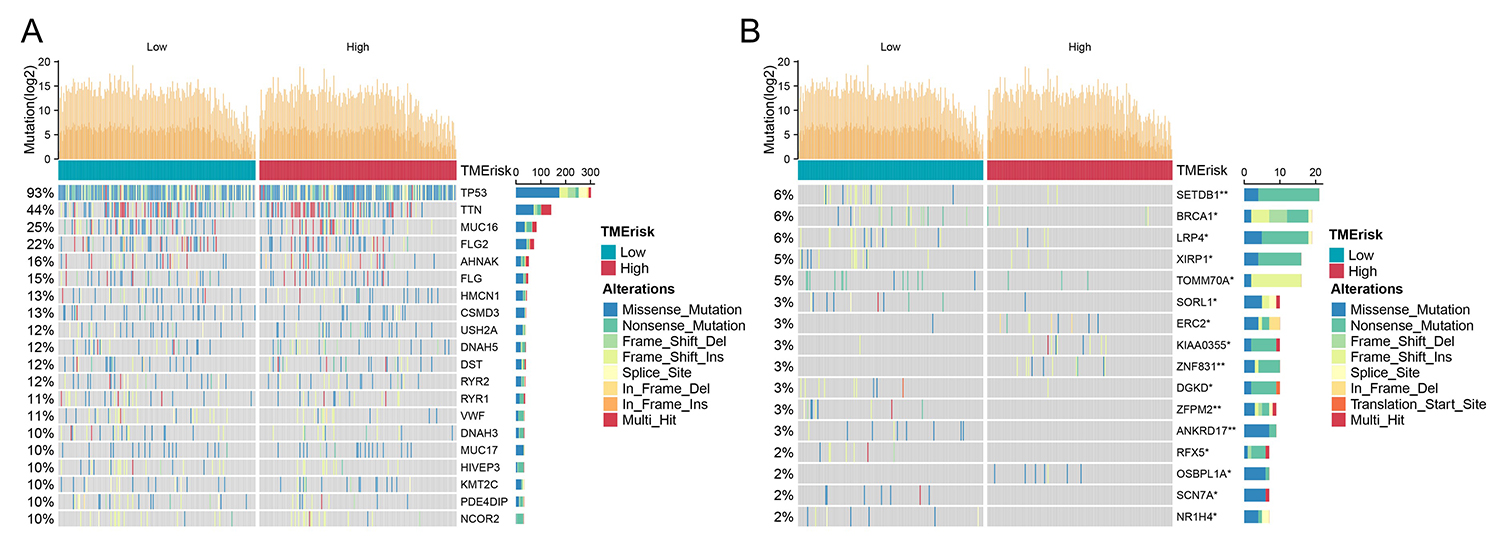

Supplement: Supplementary file 10 [file Image_9.jpeg]

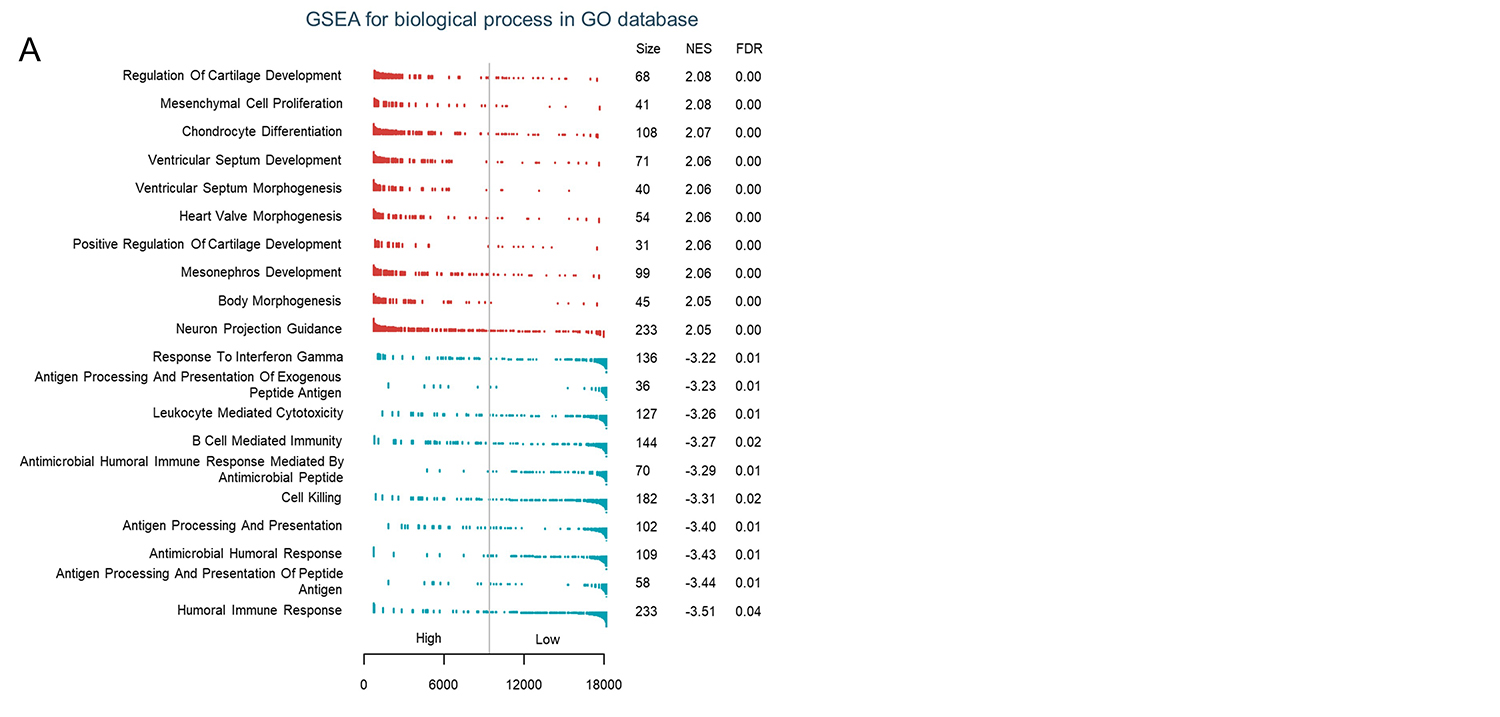

Supplement: Supplementary file 11 [file Image_10.jpeg]

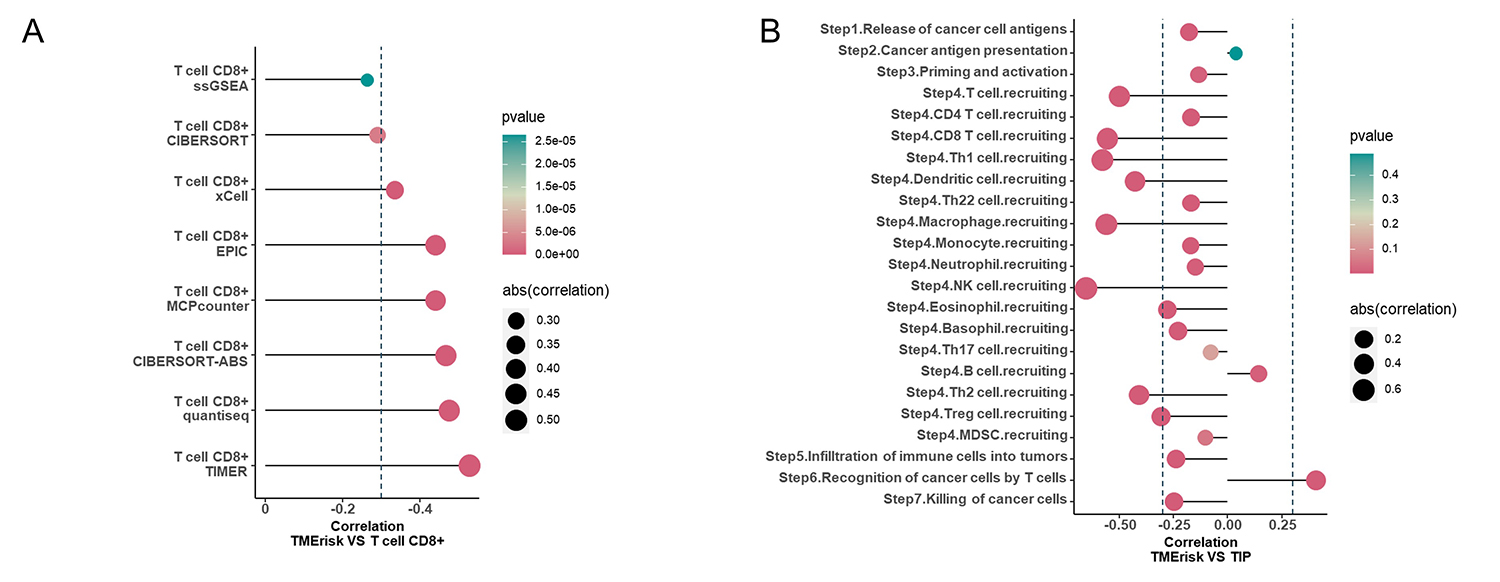

Supplement: Supplementary file 12 [file Image_11.jpeg]

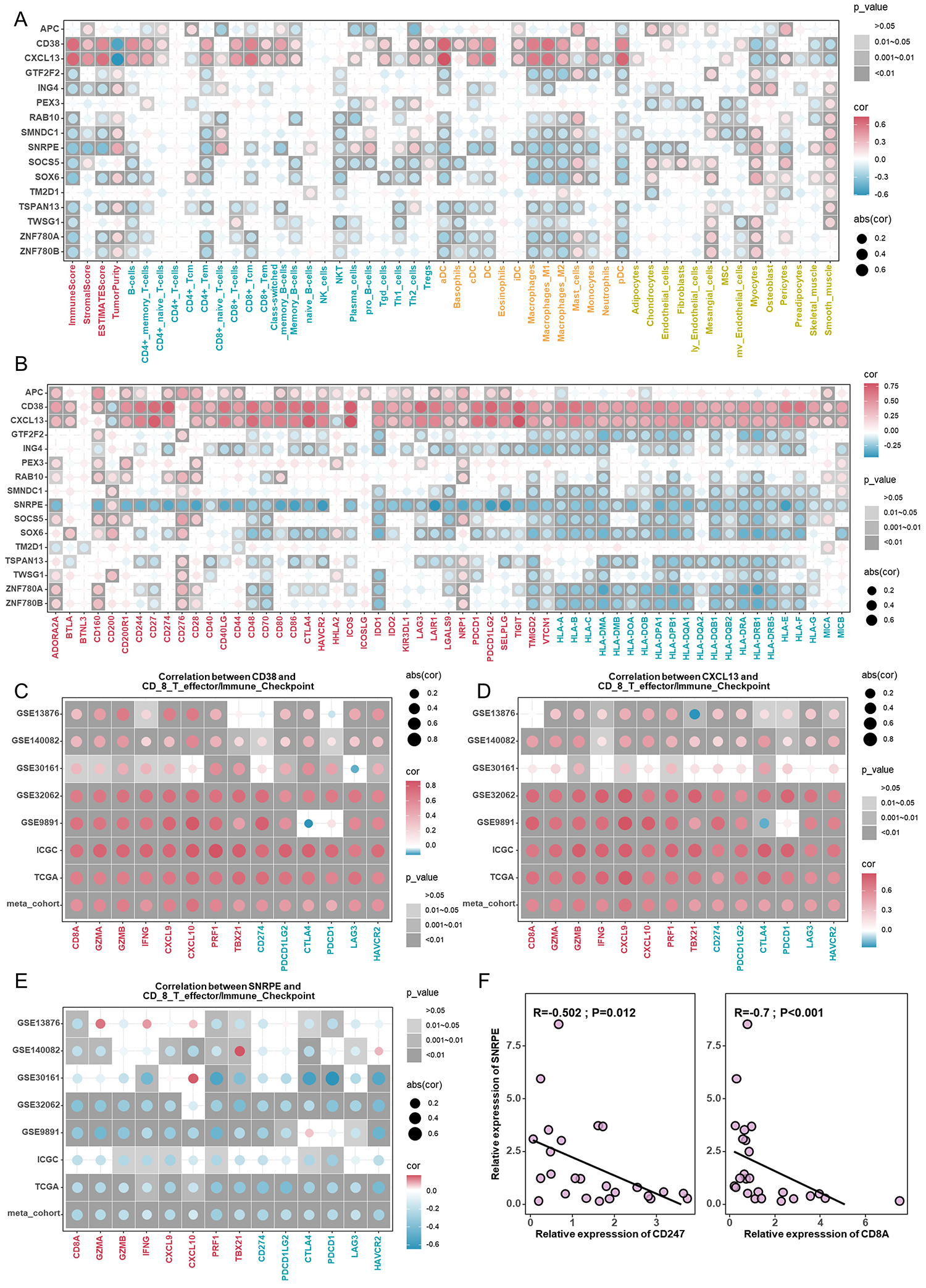

Supplement: Supplementary file 13 [file Image_12.jpeg]

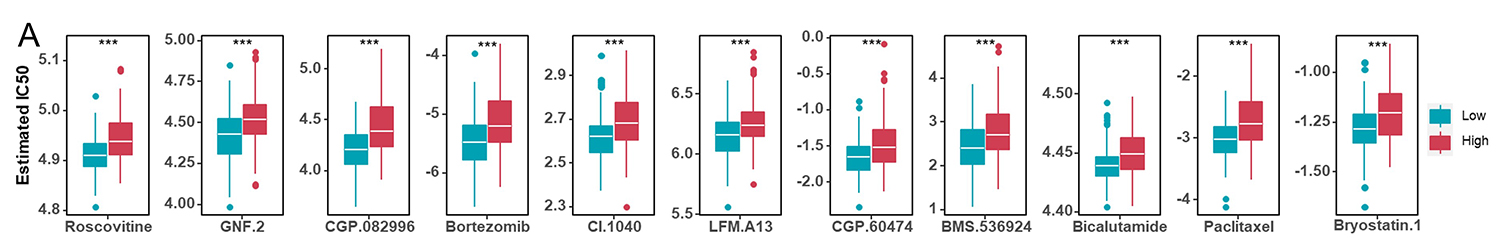

Supplement: Supplementary file 14 [file Image_13.jpeg]
